# Efficacy and safety of adding immune checkpoint inhibitors to standard chemotherapy or chemoradiotherapy for advanced or recurrent cervical cancer: a meta-analysis

**DOI:** 10.3389/fimmu.2026.1780791

**Published:** 2026-03-05

**Authors:** Le Zhou, Chuntang Sun, Peng Diao, Junchao Wang

**Affiliations:** 1 Department of Obstetrics and Gynecology, West China Second University Hospital, Sichuan University, Chengdu, China; 2 Key Laboratory of Obstetrics & Gynecologic and Pediatric Diseases and Birth Defects of Ministry of Education, West China Second University Hospital, Sichuan University, Chengdu, China; 3 Radiotherapy Department of Cancer Center, West China Second University Hospital, Sichuan University, Chengdu, China; 4 Department of Oncology, Zizhong Zizhou Hospital & Neijiang Cancer Hospital, Sichuan University, Chengdu, China; 5 Department of Radiation Oncology, Sichuan Clinical Research Center for Cancer, Sichuan Cancer Hospital and Institute, Sichuan Cancer Center, University of Electronic Science and Technology of China, Chengdu, China

**Keywords:** cervical cancer, chemoradiotherapy, chemotherapy, immune checkpoint inhibitors, overall survival, progression-free survival

## Abstract

**Background:**

Immune checkpoint inhibitors (ICIs) combined with standard chemotherapy (CT) or chemoradiotherapy (CRT) have shown promising results in recent randomized controlled trials (RCTs) for advanced or recurrent cervical cancer (CC). However, comprehensive evidence is needed to evaluate their efficacy and safety, particularly in the context of patient subgroups and immune response mechanisms. This meta-analysis aimed to synthesize data from RCTs and apply trial sequential analysis (TSA) to validate findings.

**Methods:**

We systematically searched PubMed, Web of Science, Embase, and the Cochrane Library from database inception through 19 December 2025. RCTs that evaluated the efficacy and safety of ICIs combined with CT or CRT for advanced or recurrent CC were identified and relevant data were extracted. Meta-analyses were performed to pool hazard ratios (HRs) for progression-free survival (PFS) and overall survival (OS), and risk ratios (RRs) for objective response rate (ORR) and adverse events (AEs). TSA was applied to control the risk of false-positive and false-negative findings for outcomes including PFS, OS, ORR, any grade and grade 3–5 AEs. AEs were graded based on the National Cancer Institute Common Terminology Criteria for Adverse Events (NCI-CTCAE) v.5.0.

**Results:**

5 RCTs totaling 3302 patients with CC met the inclusion criteria. The pooled analysis revealed that compared with CT or CRT (with or without placebo) the addition of ICIs to CT or CRT significantly improved PFS (HR = 0.661, 95% CI: 0.599-0.731; 95% prediction interval [PI]: 0.541-0.811) and OS (HR = 0.664, 95% CI: 0.590-0.747; 95% PI: 0.562-0.785). TSA confirmed the robustness of these findings. While the pooled ORR showed a numerical increase (RR = 1.117, 95% CI: 1.035-1.205), the 95% PI (0.888-1.404) suggested limited robustness. Subgroup analyses showed that the PFS and OS benefits were particularly pronounced in patients with a PD-L1 combined positive score (CPS) ≥ 1, while those with CPS <1 did not derive significant benefit. Safety analyses indicated that adding ICIs did not increase the risk of all-cause AEs of any grade (RR = 1.002, 95% CI: 0.996-1.008; 95% PI: 0.991-1.012), but was associated with a higher incidence of grade 3–5 AEs (RR = 1.076, 95% CI: 1.032-1.123; 95% PI: 1.021-1.144).

**Conclusion:**

Adding ICIs to CT or CRT significantly improves survival outcomes in advanced or recurrent CC, particularly in PD-L1-positive patients. However, the increased risk of grade 3–5 AEs underscores the need for vigilant toxicity monitoring and management. These findings highlight the potential of ICIs to enhance immune-mediated tumor control, offering a promising therapeutic option for selected patient populations.

## 1 Introduction

Cervical cancer (CC) remains a major global health problem, ranking among the most common malignancies and cancer-related causes of death in women worldwide, with approximately 600,000 new cases and 342,000 deaths reported annually (1–4). The burden is disproportionately borne by low- and middle-income countries, where most cases and deaths occur and access to prevention and timely treatment is limited (5, 6). The prognosis of CC varies markedly by stage: early-stage disease has favorable long−term survival, whereas locally advanced and metastatic CC are associated with substantially lower 5−year survival and short median overall survival (OS) and progression-free survival (PFS) (7–10). Standard management for locally advanced CC remains concurrent chemoradiotherapy (CRT) with platinum−based chemotherapy (CT) and brachytherapy; and systemic platinum−based regimens, with or without angiogenesis inhibition (bevacizumab), are the backbone of treatment for advanced, recurrent, or metastatic CC (11, 12). However, clinical outcomes for recurrent or metastatic CC are poor, with low second-line response rates and limited survival beyond first-line therapy, underscoring the need for more effective systemic approaches (9, 13–16).

Immune checkpoint inhibitors (ICIs) targeting programmed cell death protein 1/programmed death-ligand 1 (PD−1/PD−L1) and cytotoxic T-lymphocyte-associated protein 4 (CTLA−4) have transformed treatment across several malignancies and are biologically plausible candidates for CC given the etiologic role of high−risk human papillomavirus (HPV) and tumor immunogenicity (17, 18). Currently, several ICIs have been approved for the treatment of CC or are being investigated in clinical trials. Pembrolizumab, a monoclonal antibody targeting PD-1, has been approved for recurrent or metastatic CC with a PD-L1 combined positive score (CPS) of ≥ 1, based on the results of the KEYNOTE-826 trial (19, 20). Atezolizumab, which targets PD-L1, demonstrated improved survival outcomes when combined with platinum-based chemotherapy and bevacizumab in metastatic CC, as reported in the BEATcc trial (21). Other ICIs, such as durvalumab (anti-PD-L1) and ipilimumab (anti-CTLA-4), are currently under evaluation in clinical trials, though their roles remain investigational (22, 23). ICIs exert their antitumor effects by modulating immune responses, primarily through restoring T-cell activity and enhancing antitumor immunity. In CC, the immunosuppressive tumor microenvironment, driven by HPV oncogenic proteins (E6 and E7) and PD-L1 overexpression on tumor cells (24), makes ICIs particularly relevant. By blocking the PD-1/PD-L1 axis, ICIs prevent T-cell exhaustion and reinvigorate cytotoxic T-cell-mediated tumor killing (25). CTLA-4 inhibitors, such as ipilimumab, act earlier in the immune response by enhancing T-cell priming and proliferation through the blockade of inhibitory signals in antigen-presenting cells (26).

Although ICIs have shown promise in certain settings, the aggregate clinical value and safety profile of adding ICIs to standard CT or CRT remain incompletely defined. Individual trials vary in design, patient selection, and statistical power to evaluate subgroup effects, and prior pooled analyses have been limited by small numbers of RCTs or by mixing populations with differing disease stages (12, 27, 28). Given the heterogeneity of the available data and the therapeutic and safety implications for patients and clinicians, a comprehensive synthesis focused on randomized evidence is warranted. Accordingly, we performed a meta−analysis of phase 3 RCTs assessing the efficacy and safety of adding ICIs to standard CT or CRT for advanced or recurrent CC, and applied trial sequential analysis (TSA) to examine the robustness and sufficiency of the accumulated evidence.

## 2 Methods

### 2.1 Study design

This meta-analysis was prospectively registered in the PROSPERO database (identifier CRD420261278547; https://www.crd.york.ac.uk/prospero/). Study conduct and reporting followed the Preferred Reporting Items for Systematic Reviews and Meta−Analyses (PRISMA) 2020 guidelines (29).

### 2.2 Search strategy

Two investigators independently interrogated PubMed, Web of Science, Embase and the Cochrane Library to identify RCTs published from database inception to 19 December 2025. The search combined terms for immune−checkpoint therapy (for example, “immune checkpoint inhibitors,” “immune checkpoint blockade,” “programmed death−1,” “PD−L1,” “CTLA−4,” and agents such as pembrolizumab, nivolumab, atezolizumab, durvalumab, cadonilimab, dostarlimab and cemiplimab) with descriptors of cervical malignancy (including “uterine cervical neoplasms,” “cervical cancer,” “cervical carcinoma,” “cervical neoplasm*,” “cancer of the cervix,” and “cervix cancer”). Searches were restricted to RCTs involving human participants and were conducted without language limitations. Database−specific strategies are provided in **Supplementary File 1**. In addition, reference lists of retrieved relevant review articles were screened manually to capture any further eligible trials.

### 2.3 Study selection

Studies were considered eligible if they satisfied all of the following conditions: (1) RCT design; (2) enrollment of patients with locally advanced, persistent or recurrent, or metastatic CC; (3) experimental treatment comprising an ICI combined with standard CT or CRT; (4) comparator arm consisting of CT or CRT alone, or CT/CRT plus placebo; and (5) reporting at least one relevant endpoint, including PFS, OS, objective response rate (ORR), all-cause adverse events (AEs) of any grade, or all-cause AEs of grade 3-5. Studies were excluded if they met any of the following criteria: (1) non−randomized designs such as single−arm trials or other observational studies; (2) key data that were missing, erroneous, or unobtainable; (3) duplicate publications of the same trial; or (4) preclinical studies, conference abstracts, narrative reviews, case reports, or correspondence/letters to the editor.

### 2.4 Data extraction

Two investigators independently extracted study data using a standardized collection template. Extracted items comprised trial name and phase, first author and publication year, study sites, eligible patients, descriptions of experimental and comparator interventions, sample sizes and age distributions in each group, median duration of follow−up, and reported endpoints. When a trial yielded multiple reports, the publication providing the most complete dataset was used. PFS and OS were prespecified as the primary outcomes for pooled analyses; secondary endpoints included ORR, all-cause AEs of any grade, and all-cause grade 3–5 AEs.

### 2.5 Quality assessment

Study quality was appraised using the modified Jadad scale (30), which evaluates the adequacy of randomization, allocation concealment, blinding and the reporting of withdrawals and dropouts. Trials scoring 0–3 were classified as low quality, whereas those with scores of 4 or greater were judged high quality. Any discrepancies in ratings were resolved by consensus, with arbitration by a senior investigator when necessary.

### 2.6 Statistical analysis

Treatment effects were quantified as hazard ratios (HRs) for time−to−event endpoints and risk ratios (RRs) for binary outcomes, each accompanied by 95% confidence intervals (CIs). Between-study heterogeneity was examined using Cochran’s Q test, the I^2^ statistic and 95% prediction intervals (PIs), with I^2^ values above 50% taken to indicate meaningful heterogeneity (31, 32). In the absence of substantial heterogeneity, pooled estimates were calculated with a fixed-effect model; when heterogeneity was present, a random-effects approach (DerSimonian-Laird) was adopted (33). Prespecified subgroup analyses were constructed by aggregating the stratified PFS and OS results reported in individual trials. The stability of the meta−analytic findings was assessed with leave−one−out sensitivity analyses. Potential publication bias was evaluated by visual inspection of funnel plots and formally tested using Begg’s and Egger’s methods (34, 35). Statistical significance was set at a two-sided *p* < 0.05. All analyses were conducted using R software 4.3.2 and Stata 12.0.

### 2.7 Trial sequential analysis

To control the risk of false−positive and false−negative findings, we applied TSA to the meta−analysis (36). Time-to-event endpoints (PFS and OS) were evaluated using an *a priori* information size (APIS) framework implemented in Stata 12.0 and R 4.3.2, whereas dichotomous outcomes were assessed with TSA software v0.9.5.10 Beta to estimate the required information size (RIS) and to construct trial sequential monitoring boundaries. When the cumulative Z-curve crossed the monitoring boundary or exceeded the RIS/APIS threshold, the evidence was considered conclusive and further trials were deemed unnecessary. All RIS/APIS calculations employed prespecified parameters: a two−sided α of 0.05, 80% power (1-β = 0.80) and an assumed relative risk reduction of 15%.

## 3 Results

### 3.1 Study selection procedure

**Figure 1:**
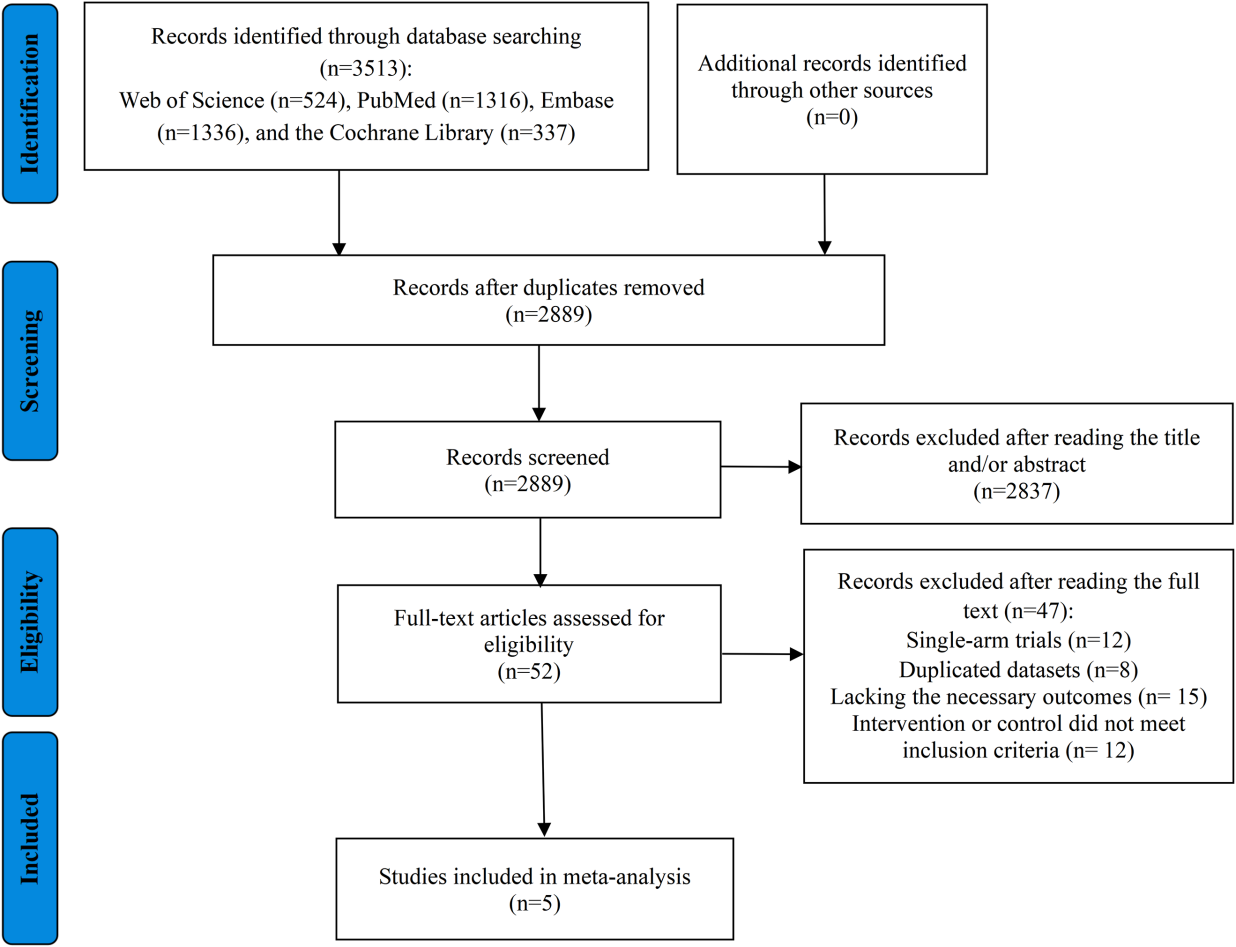
The flow diagram of studies included in this meta-analysis.

**Figure 1** summarizes the study selection process. The database search initially retrieved 3513 records; after deduplication 2889 unique records underwent title-and-abstract screening. Of these, 2837 were judged irrelevant and excluded, leaving 52 articles for full-text review. 47 of the full texts were subsequently found ineligible: 12 were single−arm studies, 8 reported duplicated patient cohorts, 15 did not provide the required outcome data, and 12 used intervention or control regimens inconsistent with our inclusion criteria. Ultimately, 5 RCTs satisfied the eligibility requirements and were included in the quantitative synthesis (21, 23, 37–39).

### 3.2 Study characteristics and quality assessment

Key study characteristics are presented in **Table 1**. The meta−analysis incorporated 5 phase-3 randomized trials (KEYNOTE−A18, CALLA, COMPASSION−16, KEYNOTE−826 and BEATcc), all reported in English between 2022 and 2025. In total, 3302 patients with advanced or recurrent CC were randomized: 1650 received an ICI in combination with standard CT or CRT, while 1652 were assigned to standard therapy with or without placebo. Investigational agents evaluated across the trials included pembrolizumab, durvalumab, cadonilimab and atezolizumab. Each study appeared in a high-impact journal and achieved modified Jadad scores of 5-7, consistent with high methodological quality. Further details of the quality assessment are provided in **Supplementary File 2**.

**Table 1:** Basic characteristics of the included RCTs.

Trial (Phase)	Study ID	Study sites	Eligible patients	Experimental group			Control group			Median follow-up duration (E/C, months)	Outcomes
				Treatment details	No. of patients	Median age (years)	Treatment details	No. of patients	Median age (years)		
KEYNOTE-A18 (Phase 3)	Lorusso et al, 2024 (37)	176 sites in 30 countries across Asia, Australia, Europe, North America, and South America	Patients (18 years or older) with newly diagnosed, high risk, locally advanced, histologically confirmed squamous cell carcinoma, adenocarcinoma, or adenosquamous carcinoma of the cervix; and had an ECOG PS score of 0 or 1	Pembrolizumab (targets PD-1, 200 mg, q3w, 5 cycles) + Chemoradiotherapy followed by pembrolizumab (400 mg, q6w, 15 cycles)	529	49 (IQR 40-57)	Placebo (q3w, 5 cycles) + Chemoradiotherapy followed by placebo (q6w, 15 cycles)	531	50 (IQR 41-59)	29.9 (IQR, 23.3-34.3)	PFS, OS, ORR, and AEs
CALLA (Phase 3)	Monk et al, 2023 (23)	105 sites in 15 countries in Europe, Asia, Africa, North America, and South America	Women aged 18 years or older with untreated histologically confirmed cervical adenocarcinoma, squamous carcinoma, or adenosquamous carcinoma; and had an ECOG PS score of 0 or 1	Durvalumab (targets PD-L1, 1500 mg, q4w) + Chemoradiotherapy for a total of 24 cycles	385	50 (IQR 41-57)	Placebo (q4w) + Chemoradiotherapy for a total of 24 cycles	385	48 (IQR 40-57)	18.5 (IQR 13.2-21.5)/18.4 (IQR 13.2-23.7)	PFS, OS, ORR, and AEs
COMPASSION-16 (Phase 3)	Wu et al, 2024 (3)	59 clinical sites in China	Women aged 18-75 years with persistent, recurrent, or metastatic (stage IVB) cervical cancer of squamous cell carcinoma, adenocarcinoma, or adenosquamous carcinoma; and had an ECOG PS score of 0 or 1	Cadonilimab (targets PD-1 and CTLA-4, 10 mg/kg, q3w, 6 cycles) + Chemotherapy (cisplatin/carboplatin + paclitaxel) followed by cadonilimab (10 mg/kg, q3w) with or without bevacizumab	222	56 (range 23-75)	Placebo (q3w, 6 cycles) + Chemotherapy (cisplatin/carboplatin + paclitaxel) followed by placebo (q3w) with or without bevacizumab	223	56 (range 23-75)	25.6 (IQR 23.6-28.0)	PFS, OS, ORR, and AEs
KEYNOTE-826 (Phase 3)	Monk et al, 2023 (38)	151 sites in 19 countries	Patients (18 years and older) with persistent, recurrent, or metastatic adenocarcinoma, adenosquamous carcinoma, or squamous cell carcinoma of the cervix; and had an ECOG PS score of 0 or 1	Pembrolizumab (targets PD-1, 200 mg, q3w) + Chemotherapy (paclitaxel plus either cisplatin or carboplatin) with or without bevacizumab	308	51 (range 25-82)	Placebo (q3w) + Chemotherapy (paclitaxel plus either cisplatin or carboplatin) with or without bevacizumab	309	50 (range 22-79)	39.1 (range, 32.1-46.5)	PFS, OS, ORR, and AEs
BEATcc (Phase 3)	Oaknin et al, 2024 (21)	92 sites in Europe, Japan, and the USA	Adult patients (≥18 years) with measurable metastatic (stage IVB), persistent, or recurrent cervical cancer of squamous cell carcinoma or adenocarcinoma subtype; and had an ECOG PS score of 0 or 1	Atezolizumab (targets PD-L1, 1200 mg, q3w) + Chemotherapy (paclitaxel plus either cisplatin or carboplatin) + Bevacizumab (15 mg/kg, q3w)	206	51 (IQR 43-60)	Chemotherapy (paclitaxel plus either cisplatin or carboplatin) + Bevacizumab (15 mg/kg, q3w)	204	52.5 (IQR 43.5-61)	32.9 (95% CI 31.2-34.6)	PFS, OS, ORR, and AEs

E, experimental group; C, control group; ECOG, Eastern Cooperative Oncology Group; PS, performance status; q3w, once every 3 weeks; IQR, interquartile range; CI, confidence interval; PFS, progression-free survival; OS, overall survival; ORR, objective response rate; AEs, adverse events; PD-1, programmed cell death protein 1; PD-L1, programmed death-ligand 1; CTLA-4, cytotoxic T-lymphocyte-associated protein 4.

### 3.3 Pooled effect and subgroup analysis of the efficacy outcomes

#### 3.3.1 PFS

**Figure 2:**
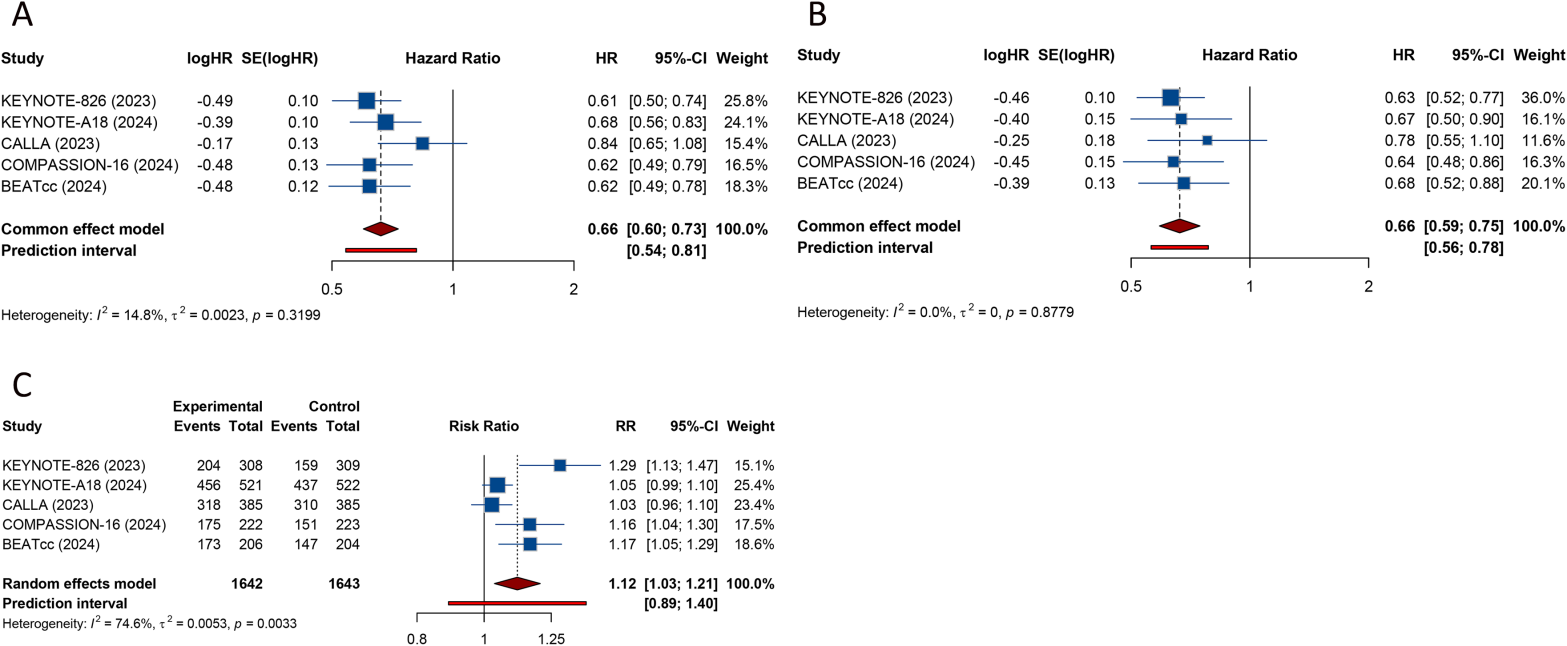
Forest plots of the efficacy outcomes of adding immune checkpoint inhibitors to standard chemotherapy or chemoradiotherapy for advanced or recurrent cervical cancer. **(A)** Progression-free survival; **(B)** Overall survival; **(C)** Objective response rate.

All 5 RCTs reported PFS in patients with advanced or recurrent CC. Low between-study heterogeneity warranted use of a fixed-effect model (I^2^ = 14.8%). The pooled analysis indicated that the addition of ICIs to standard CT or CRT significantly prolonged PFS versus standard therapy alone (or with placebo) (HR = 0.661, 95% CI: 0.599-0.731; 95% PI: 0.541-0.811) (**Table 2**; **Figure 2A**). In subgroup analyses, this PFS advantage failed to reach statistical significance in the Asian patients (HR = 0.776, 95% CI: 0.473-1.272; 95% PI: 0.005-128.612) and among patients with a PD−L1 combined positive score (CPS) <1 (HR = 0.809, 95% CI: 0.561-1.165; 95% PI: 0.076-8.619). By contrast, all other prespecified subgroups demonstrated a significant benefit from the combined regimen (all *p* < 0.05) (**Table 2**; **Supplementary Figures S1**–**S9**).

**Table 2:** Pooled effect and subgroup analysis of the primary outcomes of adding immune checkpoint inhibitors to standard chemotherapy or chemoradiotherapy for advanced or recurrent cervical cancer.

Outcomes and subgroups	Number of studies	Number of patients	Meta-analysis				Heterogeneity	
			HR	95% CI	*p* value	95% PI	I^2^, Tau^2^	*p* value
PFS								
**Overall**	5	3302	0.661	0.599-0.731	<0.001	0.541-0.811	14.8%, 0.002	0.320
Therapeutic targets								
PD-1	2	1677	0.643	0.558-0.740	<0.001	0.258-1.603	0%, 0	0.450
PD-L1	2	1180	0.718	0.534-0.967	0.029	0.038-13.661	66.6%, 0.031	0.084
PD-1 and CTLA-4	1	445	0.620	0.485-0.792	<0.001			
Age								
< 65 years	5	2838	0.675	0.606-0.752	<0.001	0.548-0.837	13.2%, 0.002	0.330
≥ 65 years	5	464	0.590	0.449-0.775	<0.001	0.289-1.232	28.9%, 0.040	0.229
Race								
White	3	1102	0.654	0.557-0.768	<0.001	0.460-0.931	0%, 0	0.573
Asian	2	745	0.776	0.473-1.272	0.314	0.005-128.612	76.2%, 0.098	0.040
Disease status								
Metastatic	3	603	0.760	0.623-0.928	0.007	0.491-1.177	0%, 0	0.666
Non-metastatic	3	869	0.552	0.467-0.652	<0.001	0.383-0.796	0%, 0	0.675
ECOG performance status								
0	4	1550	0.644	0.557-0.745	<0.001	0.508-0.816	0%, 0	0.500
1	4	977	0.635	0.540-0.747	<0.001	0.488-0.827	0%, 0	0.830
PD-L1 combined positive score								
<1	2	185	0.809	0.561-1.165	0.254	0.076-8.619	0%, 0	0.491
1 to <10	2	543	0.638	0.515-0.792	<0.001	0.158-2.575	0%, 0	0.776
≥10	2	497	0.523	0.419-0.653	<0.001	0.124-2.209	0%, 0	0.939
Concomitant bevacizumab								
Yes	2	654	0.666	0.473-0.938	0.020	0.023-19.282	64.2%, 0.040	0.095
No	2	408	0.569	0.383-0.846	0.005	0.012-27.520	63.5%, 0.052	0.098
Chemotherapy backbone								
Cisplatin	2	435	0.613	0.486-0.774	<0.001	0.135-2.781	0%, 0	0.417
Carboplatin	2	420	0.653	0.514-0.829	0.001	0.139-3.068	0%, 0	0.413
Previous chemoradiotherapy								
Yes	2	478	0.550	0.442-0.684	<0.001	0.134-2.254	0%, 0	0.999
No	2	377	0.741	0.577-0.952	0.019	0.146-3.772	0%, 0	0.795
OS								
**Overall**	5	3302	0.664	0.590-0.747	<0.001	0.562-0.785	0%, 0	0.878
Therapeutic targets								
PD-1	2	1677	0.642	0.545-0.756	<0.001	0.223-1.850	0%, 0	0.733
PD-L1	2	1180	0.715	0.580-0.882	0.002	0.184-2.781	0%, 0	0.537
PD-1 and CTLA-4	1	445	0.640	0.478-0.857	0.003			
Age								
< 65 years	4	2154	0.643	0.561-0.737	<0.001	0.515-0.802	0%, 0	0.731
≥ 65 years	4	378	0.689	0.505-0.941	0.019	0.256-1.930	36.7%, 0.060	0.192
Race								
White	3	1102	0.710	0.589-0.855	<0.001	0.412-1.242	15.7%, 0.005	0.305
All others	3	847	0.591	0.475-0.735	<0.001	0.366-0.953	0%, 0	0.548
Disease status								
Metastatic	3	603	0.795	0.637-0.994	0.044	0.488-1.297	0%, 0	0.802
Non-metastatic	3	869	0.573	0.480-0.684	<0.001	0.389-0.845	0%, 0	0.515
ECOG performance status								
0	4	1550	0.679	0.569-0.810	<0.001	0.510-0.905	0%, 0	0.838
1	4	977	0.635	0.529-0.761	<0.001	0.473-0.852	0%, 0	0.876
PD-L1 combined positive score								
<1	2	185	0.819	0.552-1.213	0.319	0.064-10.488	0%, 0	0.761
1 to <10	2	543	0.658	0.520-0.832	0.001	0.143-3.017	0%, 0	0.705
≥10	2	497	0.605	0.474-0.773	<0.001	0.124-2.959	0%, 0	0.572
Concomitant bevacizumab								
Yes	2	654	0.672	0.538-0.839	0.001	0.047-10.120	40.2%, 0.021	0.196
No	2	408	0.603	0.471-0.772	<0.001	0.069-5.164	19.7%, 0.008	0.265
Chemotherapy backbone								
Cisplatin	2	435	0.594	0.332-1.063	0.080	0.002-235.786	75.4%, 0.134	0.044
Carboplatin	2	420	0.700	0.533-0.920	0.011	0.031-15.357	40.2%, 0.027	0.196
Previous chemoradiotherapy								
Yes	2	478	0.585	0.455-0.752	<0.001	0.114-2.988	0%, 0	0.651
No	2	377	0.801	0.599-1.071	0.135	0.122-5.272	0%, 0	0.680

PFS, progression-free survival; PD-1, programmed cell death protein 1; PD-L1, programmed death-ligand 1; CTLA-4, cytotoxic T-lymphocyte-associated protein 4; ECOG, Eastern Cooperative Oncology Group; OS, overall survival.

Bold values indicate section headers.

#### 3.3.2 OS

5 studies evaluated the effect of adding ICIs to CT or CRT on OS in advanced or recurrent. Due to the lack of significant heterogeneity among the studies (I^2^ = 0%), a fixed-effects model was used for the analysis. The combined analysis demonstrated a statistically significant OS advantage for regimens incorporating ICIs versus CT or CRT alone (or with placebo) (HR = 0.664, 95% CI: 0.590-0.747; 95% PI: 0.562-0.785) (**Table 2**; **Figure 2B**). The subgroup analyses showed that ICIs added to CT or CRT failed to produce a significant improvement in OS only among patients with PD-L1 CPS <1 (HR = 0.819, 95% CI: 0.552-1.213; 95% PI: 0.064-10.488), those treated with a cisplatin chemotherapy backbone (HR = 0.594, 95% CI: 0.332-1.063; 95% PI: 0.002-235.786), and patients without prior CRT (HR = 0.801, 95% CI: 0.599-1.071; 95% PI: 0.122-5.272). In all other subgroups, the addition of ICIs was associated with a significant OS benefit (all *p* < 0.05) (**Table 2**; **Supplementary Figures S10**-**S18**).

#### 3.3.3 ORR

Each of the 5 trials reported ORR for ICIs administered alongside CT or CRT in cervical carcinoma. Because study heterogeneity was substantial (I^2^ = 74.6%), a random−effects model was applied. The combined estimate showed that compared with standard CT or CRT alone or combined with placebo, the addition of ICIs to standard CT/CRT significantly increased ORR (RR = 1.117, 95% CI: 1.035-1.205). However, the 95% PI (0.888-1.404) crossed 1, indicating a degree of uncertainty regarding the robustness of this effect (**Table 2**; **Figure 2C**). Subgroup analysis based on therapeutic targets revealed that the combination therapy showed a significant improvement in ORR only in the subgroup targeting both PD-1 and CTLA-4 from a single study (RR = 1.164, 95% CI: 1.039-1.304), while no significant improvement was observed in subgroups with PD-1 or PD-L1 as the therapeutic targets (all *p* > 0.05) (**Table 3**; **Supplementary Figure 19**).

**Table 3:** Pooled effect and subgroup analysis of the secondary outcomes of adding immune checkpoint inhibitors to standard chemotherapy or chemoradiotherapy for advanced or recurrent cervical cancer.

Outcomes	Number of studies	Number of patients	Meta-analysis				Heterogeneity	
			RR	95% CI	*p* value	95% PI	I^2^, Tau^2^	*p* value
ORR								
**Overall**	5	3285	1.117	1.035-1.205	0.005	0.888-1.404	74.6%, 0.005	0.003
**Therapeutic targets**								
PD-1	2	1660	1.151	0.916-1.447	0.227	0.096-13.797	90.2%, 0.025	0.001
PD-L1	2	1180	1.086	0.959-1.231	0.194	0.300-3.941	75.6%, 0.006	0.043
PD-1 and CTLA-4	1	445	1.164	1.039-1.304	0.009			
All-cause AEs of any grade								
**Overall**	5	3292	1.002	0.996-1.008	0.562	0.991-1.012	15.4%, <0.001	0.316
Therapeutic targets								
PD-1	2	1674	1.005	0.998-1.012	0.184	0.957-1.056	8.4%, <0.001	0.296
PD-L1	2	1173	1.000	0.986-1.015	0.983	0.912-1.095	0%, 0	0.611
PD-1 and CTLA-4	1	445	0.996	0.983-1.008	0.489			
Specific events								
Anemia	5	3292	1.040	0.984-1.099	0.163	0.861-1.240	41.1%, 0.003	0.147
Vomiting	5	3292	1.007	0.905-1.121	0.896	0.759-1.370	29.6%, 0.007	0.224
Diarrhea	5	3292	1.092	0.936-1.274	0.265	0.704-1.693	67.1%, 0.019	0.016
Constipation	5	3292	1.028	0.918-1.152	0.630	0.795-1.348	21.5%, 0.005	0.278
Nausea	5	3292	0.989	0.927-1.055	0.741	0.901-1.080	0%, 0	0.557
Decreased appetite	5	3292	1.186	1.033-1.360	0.015	0.757-1.930	43.0%, 0.019	0.135
Urinary tract infection	5	3292	0.968	0.860-1.089	0.588	0.775-1.221	11.8%, 0.003	0.339
Fatigue	4	2847	0.926	0.800-1.071	0.298	0.611-1.416	28.0%, 0.009	0.244
Hypothyroidism	4	2676	2.988	2.397-3.724	<0.001	2.078-4.250	0%, 0	0.793
Neutropenia	4	2847	1.134	0.993-1.295	0.063	0.903-1.384	0%, 0	0.399
Platelet count decreased	4	2676	0.954	0.827-1.101	0.521	0.760-1.225	1.2%, <0.001	0.386
White blood cell count decreased	4	2676	0.903	0.817-0.998	0.046	0.780-1.051	0%, 0	0.874
Alanine aminotransferase increased	4	2676	1.109	0.808-1.522	0.521	0.437-2.816	60.3%, 0.060	0.056
All-cause AEs of grade 3-5								
Overall	5	3292	1.076	1.032-1.123	0.001	1.021-1.144	0%, 0	0.690
Therapeutic targets								
PD-1	2	1674	1.108	1.050-1.170	<0.001	0.780-1.570	0%, 0	0.688
PD-L1	2	1173	1.028	0.940-1.126	0.544	0.597-1.793	0%, 0	0.674
PD-1 and CTLA-4	1	445	1.063	0.976-1.157	0.160			
Specific events								
Anemia	5	3292	1.092	0.851-1.403	0.489	0.529-2.256	67.7%, 0.052	0.015
Vomiting	5	3292	0.869	0.487-1.553	0.636	0.379-2.048	0%, 0	0.680
Diarrhea	5	3292	1.347	0.913-1.989	0.134	0.489-3.167	15.8%, 0.052	0.314
Constipation	5	3292	0.707	0.225-2.225	0.553	0.052-10.340	0%, 0	0.568
Nausea	5	3292	1.112	0.653-1.895	0.696	0.457-2.642	0%, 0	0.697
Decreased appetite	5	3292	3.287	1.370-7.886	0.008	0.895-11.174	0%, 0	0.915
Urinary tract infection	5	3292	1.113	0.824-1.503	0.485	0.727-1.704	0%, 0	0.983
Fatigue	4	2847	0.787	0.457-1.356	0.388	0.335-2.024	0%, 0	0.688
Hypothyroidism	4	2676	5.927	0.717-49.016	0.099	–	0%, 0	0.863
Neutropenia	4	2847	1.095	0.793-1.510	0.582	0.429-2.791	56.1%, 0.060	0.077
Platelet count decreased	4	2676	1.192	0.851-1.669	0.307	0.685-2.066	0%, 0	0.419
White blood cell count decreased	4	2676	0.862	0.733-1.014	0.072	0.661-1.115	0%, 0	0.729
Alanine aminotransferase increased	4	2676	1.200	0.602-2.394	0.605	0.167-7.900	16.0%, 0.157	0.312

ORR, objective response rate; PD-1, programmed cell death protein 1; PD-L1, programmed death-ligand 1; CTLA-4, cytotoxic T-lymphocyte-associated protein 4; AEs, adverse events.

Bold values indicate section headers.

### 3.4 Meta-analysis of the safety outcomes

#### 3.4.1 All-cause AEs of any grade

**Figure 3:**
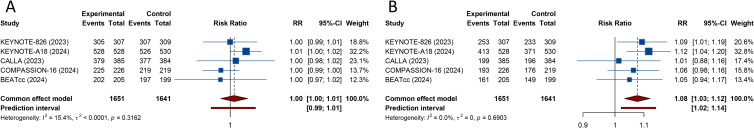
Forest plots of the safety outcomes of adding immune checkpoint inhibitors to standard chemotherapy or chemoradiotherapy for advanced or recurrent cervical cancer. **(A)** All-cause adverse events (AEs) of any grade; **(B)** All-cause AEs of grade 3-5.

5 randomized trials reported the incidence of all−cause AEs of any grade in experimental and control arms. Between-study heterogeneity was low (I^2^ = 15.4%), permitting use of a fixed−effect model for pooling. The combined analysis revealed no statistically significant difference in the overall risk of any grade AEs between patients receiving ICIs in combination with CT or CRT and those treated with CT or CRT alone or with placebo (RR = 1.002, 95% CI: 0.996-1.008; 95% PI: 0.991-1.012) (**Table 3**; **Figure 3A**). Similarly, subgroup analysis based on therapeutic targets suggested that no increase in the overall risk of AEs was observed across the subgroups (all *p* > 0.05) (**Table 3**; **Supplementary Figure 20**).

Across the included trials, the most commonly reported events were anemia, vomiting, diarrhea, constipation, nausea, decreased appetite, urinary tract infection (UTI), fatigue, hypothyroidism, neutropenia, platelet count (PLT) decreased, white blood cell count (WBC) decreased, and alanine aminotransferase (ALT) increased. Meta-analyses of individual AEs showed that the addition of ICIs was associated with a significantly higher risk of decreased appetite (RR = 1.186, 95% CI: 1.033-1.360; 95% PI: 0.757-1.930) and hypothyroidism (RR = 2.988, 95% CI: 2.397-3.724; 95% PI: 2.078-4.250), and a significantly lower risk of white blood cell count decreased (RR = 0.903, 95% CI: 0.817-0.998; 95% PI: 0.780-1.051). There were no significant differences between groups for the other AEs (all *p* > 0.05) (**Table 3**; **Supplementary Figures S21**, **S22**).

#### 3.4.2 All-cause AEs of grade 3-5

5 studies assessed the prevalence of all-cause AEs of grade 3-5. Due to the lack of notable heterogeneity among the studies (I^2^ = 0%), a fixed-effects model was employed for the analysis. The combined results indicated that the addition of ICIs to CT or CRT resulted in a higher incidence of grade 3–5 AEs compared to the control group (RR = 1.076, 95% CI: 1.032-1.123; 95% PI: 1.021-1.144) (**Table 3**; **Figure 3B**). Further subgroup analysis stratified by therapeutic targets suggested that the combination therapy was associated with an overall increased risk of AEs only in the subgroup targeting PD-1 (RR = 1.108, 95% CI: 1.050-1.170; 95% PI: 0.780-1.570), whereas no significant increase was observed in the other subgroups (all *p* > 0.05) (**Table 3**; **Supplementary Figure 23**).

Across the 5 RCTs, the most frequently observed grade 3–5 AEs included anemia, vomiting, diarrhea, constipation, nausea, decreased appetite, UTI, fatigue, hypothyroidism, neutropenia, PLT decreased, WBC decreased, and ALT increased. Meta-analysis revealed that the addition of ICIs to CT or CRT was associated only with an increased risk of decreased appetite (RR = 3.287, 95% CI: 1.370-7.886; 95% PI: 0.895-11.174). The incidence of the other listed AEs did not differ appreciably between groups (all *p* > 0.05) (**Table 3**; **Supplementary Figures S24**, **S25**).

### 3.5 Trial sequential analysis results

**Figure 4:**
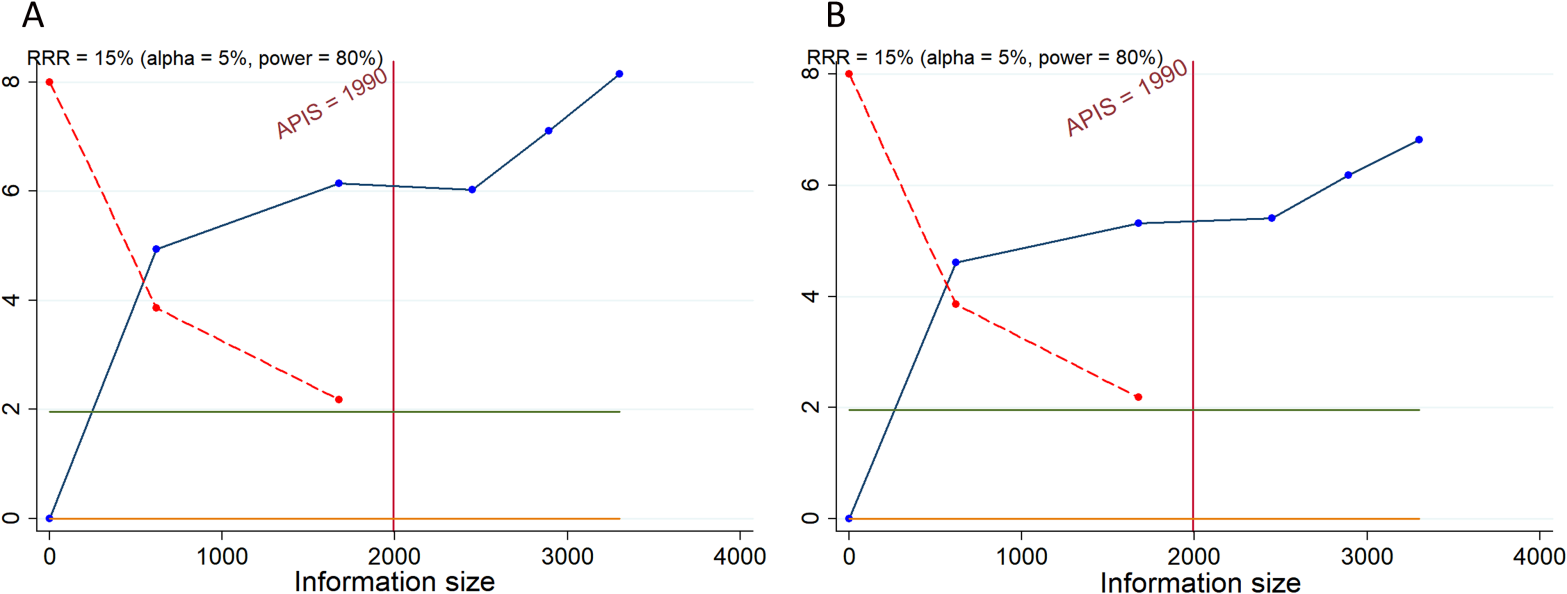
Trial sequential analysis of progression-free survival **(A)** and overall survival **(B)** of adding immune checkpoint inhibitors to standard chemotherapy or chemoradiotherapy for advanced or recurrent cervical cancer. Red inward-sloping line to the left represents trial sequential monitoring boundary. Blue line represents evolution of cumulative Z-score. Horizontal green lines represent the conventional boundaries for statistical significance. Horizontal orange lines represent the null line. Heterogeneity-adjusted a priori information size (APIS) to demonstrate or reject 15% relative risk reduction (RRR) of mortality risk (with a two-sided alpha of 5% and a statistical power of 80%) is 1990 patients for PFS and OS (vertical red line). Cumulative Z-curve crossing the trial sequential monitoring boundary or the APIS boundary provides firm evidence of effect.

**Figure 5:**
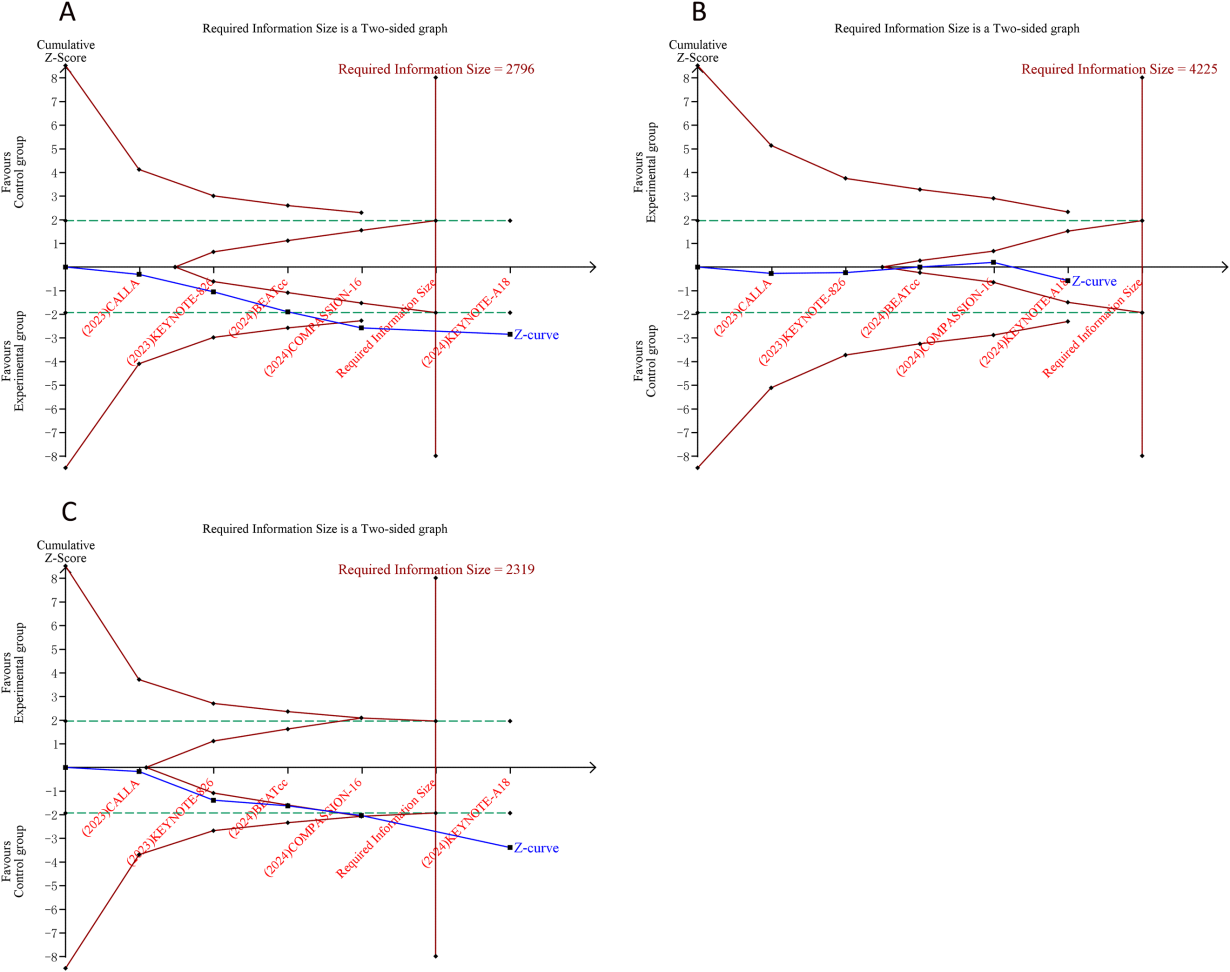
Trial sequential analysis of objective response rate **(A)**, all-cause adverse events (AEs) of any grade **(B)**, and all-cause AEs of grade 3-5 **(C)** after adding immune checkpoint inhibitors to standard chemotherapy or chemoradiotherapy for advanced or recurrent cervical cancer. Uppermost and lowermost red curves represent trial sequential monitoring boundary lines for benefit and harm, respectively. Inner red lines represent the futility boundary. Blue line represents evolution of cumulative Z-score. Horizontal green lines represent the conventional boundaries for statistical significance. The heterogeneity-adjusted required information size (RIS) was calculated to assess whether a 15% relative risk reduction (RRR) in incidence could be confirmed or excluded, with a two-sided alpha of 5% and a statistical power of 80%. Cumulative Z-curve crossing the trial sequential monitoring boundary or the RIS boundary provides firm evidence of effect.

An APIS of 1990 was determined for the TSA of PFS and OS. The cumulative Z-curves for both PFS and OS rose above the APIS line and crossed the sequential monitoring boundary, indicating these endpoints yield firm, conclusive evidence and that additional trials are unlikely to change the conclusions (**Figure 4**). Similarly, when examining dichotomous outcomes, the Z-curve for ORR and for grade 3–5 all-cause AEs surpassed both the sequential monitoring threshold and the RIS, suggesting these findings are relatively stable. In contrast, the Z-curve for all-cause AEs of any grade failed to cross either the RIS or the monitoring boundary, implying the evidence for this outcome remains uncertain and might be a false-positive observation (**Figure 5**).

### 3.6 Sensitivity analysis and publication bias

A leave-one-out sensitivity analysis was conducted to evaluate the impact of individual trials on the pooled efficacy and safety estimates. Sequential removal of each individual study did not meaningfully change the statistical significance of the pooled estimates, supporting the stability of the results (**Supplementary Figure 26**). Begg’s and Egger’s tests suggested potential publication bias for ORR (Begg’s: *p* = 0.086, Egger’s: *p* = 0.028) and all-cause AEs of grade 3-5 (Begg’s: *p* = 0.027, Egger’s: *p* = 0.028). However, applying the trim-and-fill correction produced adjusted pooled estimates that were concordant with the original analyses in both direction and significance, implying that the existence of publication bias is unlikely to have altered the final conclusions. No publication bias was detected for the other outcomes. Corresponding funnel plots are provided in **Supplementary Figure 27**.

## 4 Discussion

The integration of ICIs into the management of advanced or recurrent CC has progressed rapidly, yielding encouraging signals alongside unresolved uncertainties. Although multiple contemporary phase 3 studies have reported clinically meaningful improvements with PD−1/PD−L1 inhibitors added to standard systemic therapy (37, 40), other large trials did not meet primary endpoints (23), reflecting heterogeneity in efficacy and warranting careful interpretation. Our study conducted a pooled analysis of high-quality RCTs and found that the addition of ICIs to standard CT or CRT, compared with CT or CRT alone or in combination with placebo, significantly improved PFS, OS, and ORR in patients with advanced or recurrent CC. Furthermore, the addition of ICIs did not increase the risk of all-cause AEs of any grade but was associated with a slight increase in the risk of grade 3–5 all-cause AEs, notably a significant rise in the incidence of decreased appetite.

Compared to earlier meta-analyses assessing ICIs in CC, our research offers a more thorough evaluation by integrating TSA. This approach helps reduce the likelihood of false-positive results caused by limited data. Luo et al. conducted a meta-analysis that focused solely on PD-1/PD-L1 inhibitors and observed notable improvements in PFS and OS (41). However, their study did not consider PIs, which are essential for evaluating the consistency and applicability of the results. Additionally, although certain studies did not meet our inclusion criteria, they still provide meaningful insights into specific outcomes. The recent phase I randomized trial (NRG-GY017) involving patients with locally advanced cervical cancer (LACC) sought to identify the best sequence for administering atezolizumab alongside CRT (42). The results revealed favorable 2-year disease-free survival (DFS) in both groups: one receiving neoadjuvant atezolizumab prior to CRT and continued during CRT, and the other receiving atezolizumab only during CRT. Furthermore, administering atezolizumab before CRT was shown to be both safe and linked to favorable clinical and immunological outcomes (42). These findings suggest that further exploration of the optimal sequencing of ICIs with standard CT or CRT in clinical trials is warranted.

Biological and mechanistic evidence supports combining ICIs with cytotoxic CT, anti-angiogenic agents, or CRT to achieve improved survival outcomes. Tumor PD-L1 expression and the immunogenic properties of HPV-associated tumors are plausible biological factors underlying the sensitivity of CC to PD-1/PD-L1 blockade (43–45). CT remains the cornerstone of treatment for advanced CC; however, emerging evidence suggests that combining CT with ICIs may confer additional survival benefits in select patient populations (46). This survival improvement likely stems from the complementary mechanisms of action between CT and ICIs (47). Beyond its well-established cytotoxic effects, CT has been shown to induce immunogenic cell death, a process that releases tumor-associated antigens and primes the adaptive immune response, particularly through the activation of effector T cells (48). This immunomodulatory effect enhances tumor antigen recognition, thereby creating a tumor microenvironment more amenable to ICI-mediated immune activation (49). Additionally, the combination of CRT with ICIs is gaining significant attention in the management of CC. This combination has been demonstrated to substantially enhance immune activation, as evidenced by the upregulation of immune markers and the expansion of central and effector memory T cells, underscoring its potential to modulate the immune landscape of the tumor (50). The PRIMMO phase 2 clinical trial evaluated the effectiveness of pembrolizumab in conjunction with stereotactic body radiotherapy (SBRT) and immunomodulatory agents in patients with recurrent or metastatic CC. The trial reported a PFS duration of 4.1 weeks, indicating that these therapies maintain consistent and durable antitumor responses (51).

The ORR reported in the included trials evaluating the combination of ICIs with CT or CRT for advanced or recurrent CC ranged from 66.2% to 87.5%. In a phase 2 study involving 27 patients with recurrent or metastatic CC, the combination of sintilimab and CT achieved an ORR of 44.4% (52). The enhanced therapeutic effect observed in these combinations may be attributed to the complementary mechanisms of action: CT or CRT promotes immunogenic cell death, releasing tumor antigens, while ICIs prevent T-cell exhaustion, thereby restoring immune activity (53). Nevertheless, despite the relatively high response rates, complete responses were not achieved in all cases, suggesting that variables such as the tumor microenvironment and individual immune profiles play a critical role in influencing treatment outcomes (54). Additionally, the precise scheduling, dose and modality of local therapy that best synergize with immune modulation remain undefined. Differences between PD−1 and PD−L1 antagonists, such as receptor engagement on T cells versus ligand blockade within the tumor microenvironment, have been posited as one potential explanation for discordant trial results (55). Variations in trial populations and differences in radiation dosing strategies may further explain the divergent findings between trials like KEYNOTE-A18 and CALLA (56–58). These mechanistic and design−related nuances emphasize that combination strategies are not interchangeable and that optimization will require both translational investigation and prospective clinical testing.

ICIs that target distinct pathways exhibit diverse mechanisms of action, potentially leading to varying clinical outcomes. PD-1 inhibitors function by disrupting the interaction between PD-1, expressed on dysfunctional T cells, and its ligands PD-L1 and PD-L2, thereby reactivating T-cell responses and boosting anti-tumor immunity (59). Conversely, PD-L1 inhibitors operate within the tumor microenvironment, preventing PD-L1 from binding to PD-1 and CD80, which may reduce tumor-driven immune suppression while preserving PD-L2 signaling (60). These mechanistic differences likely contribute to observed disparities in therapeutic efficacy and toxicity profiles across clinical trials. CTLA-4 inhibitors, by targeting an alternative immune checkpoint pathway, block the inhibitory signals mediated by CTLA-4 on both regulatory and conventional T cells, thereby enhancing T-cell priming and proliferation (61). Although CTLA-4 inhibitors have shown clinical benefit in melanoma (61), their impact in CC remains less well-defined, potentially due to differences in tumor immunogenicity and characteristics of the tumor microenvironment. Emerging evidence indicates that the differences in immune checkpoint engagement may also shape the dynamics of immune responses (62). These mechanistic distinctions highlight the need to customize ICI approaches to the unique immune landscape of CC, which is marked by heterogeneous antigen expression and diverse mechanisms of immune evasion (63).

Biomarker-driven patient selection is consistently highlighted in the literature as a critical determinant of therapeutic benefit. Among these, PD-L1 expression, typically quantified using the CPS, has been shown to correlate with greater relative benefit across multiple studies and serves as the basis for current regulatory approvals in specific clinical settings (40, 64). Analyses, including pooled exploratory assessments, have demonstrated that patients with PD-L1-positive tumors (e.g., CPS ≥1 or higher thresholds) experience more pronounced treatment benefits (64), whereas those with low or absent PD-L1 expression derive no significant therapeutic benefit (38, 39). Our subgroup analysis further indicated that in CC patients with a PD-L1 CPS of ≥1, the addition of ICIs to CT or CRT significantly improved PFS and OS. In contrast, for patients with a CPS <1, the combination of ICIs did not confer any improvement in PFS or OS. Beyond PD-L1, genomic biomarkers such as mismatch−repair deficiency (dMMR)/microsatellite instability−high (MSI−H) and tumor mutational burden (TMB) have emerged as potential predictive factors, particularly in uterine and related gynecologic malignancies. Preliminary evidence indicates that these biomarkers may also stratify benefit for PD−1 blockade in CC subgroups (65, 66). Among the trials included in our analysis, three specifically employed the PD-L1 IHC 22C3 pharmDx assay to evaluate PD-L1 expression, categorizing it based on CPS thresholds of <1, 1 to <10, or ≥10 (37–39). In contrast, the CALLA trial utilized the VENTANA PD-L1 (SP263) assay to assess PD-L1 expression using the tumor area positivity (TAP) score, with TAP ≥1% serving as the cutoff (23). Exploratory biomarker analyses for the BEATcc trial, which include evaluating PD-L1 status, are scheduled and will be presented in a separate report (21). Therefore, more stratified trials employing standardized biomarker assays are needed to determine which biomarker-defined subgroups of patients with CC derive the greatest therapeutic benefit from combining ICIs with CT or CRT.

Safety and tolerability of ICI−containing regimens are central to their clinical adoption. Across randomized and single−arm series, ICIs have been associated with immune−related AEs, most commonly endocrine toxicities such as hypothyroidism, along with dermatologic, hepatic, and gastrointestinal events (11, 67, 68). These events are often manageable with treatment interruption, corticosteroids, or hormone replacement therapy when indicated (12). Some phase 3 studies and pooled analyses report a modest increase in grade 3–5 AEs when ICIs are added to CT or CRT (41), although the incidence of treatment discontinuation has not uniformly increased and fatal toxicities remain uncommon. Our study identified that across the 5 included RCTs, the most commonly reported AEs of any grade and grade 3–5 included anemia, vomiting, diarrhea, constipation, nausea, decreased appetite, UTI, fatigue, hypothyroidism, neutropenia, PLT decreased, WBC decreased, and ALT increased. Notably, the risks of decreased appetite, regardless of any grade or grade 3-5, were significantly higher in patients receiving ICIs combined with CT or CRT compared with the control group. Among other AEs of any grade, the incidence of hypothyroidism was higher in the experimental group, whereas the risk of decreased WBC was slightly lower compared with the control group.

Hypothyroidism, a frequently observed endocrine immune-related adverse event (irAE), is believed to arise from immune-mediated damage to thyroid tissue, potentially driven by the activation of autoreactive T cells (69). The development of irAEs is closely associated with the therapeutic mechanism of ICIs, which enhance anti-tumor immunity by disrupting inhibitory immune pathways but can also provoke immune dysregulation and unintended tissue damage. By targeting the PD-1/PD-L1 axis, PD-1 and PD-L1 inhibitors restore T-cell functionality, thereby amplifying anti-tumor responses. However, this immune reactivation may inadvertently extend to healthy tissues, leading to irAEs across multiple organ systems (70). In patients undergoing ICI therapy in combination with CT or CRT, the observed increase in decreased appetite is likely multifactorial, involving both immune-mediated mechanisms and systemic inflammatory processes. Elevated levels of pro-inflammatory cytokines, which are induced during ICI treatment, have been implicated in the pathogenesis of cancer-associated anorexia and cachexia (71). Although there were no significant differences in the incidence of most AEs between the two groups, our statistically significant findings support the careful adoption of ICI combinations in settings with established clinical benefit, accompanied by rigorous toxicity monitoring and well-defined management protocols. To enhance the safety and tolerability of incorporating ICIs into CT or CRT, future research should prioritize the development of strategies aimed at mitigating AEs, including dose optimization and the implementation of prophylactic approaches.

This research is subject to several limitations. First, discrepancies in patient demographics, combination therapies, treatment durations, and dosages across the included studies may have contributed to outcome heterogeneity. Nevertheless, the low heterogeneity observed in the majority of pooled outcomes enhances the reliability and clinical significance of this study. Second, publication bias was identified in the analysis of ORR and grade 3–5 AEs, potentially leading to an overestimation of the results. The underreporting of smaller studies with negative or null findings may have further skewed the pooled estimates. Although we employed the trim-and-fill method to address this issue, the presence of publication bias remains a notable limitation of our study. Furthermore, the number of studies included in certain subgroup analyses was limited, reducing the statistical power and generalizability of these findings. As a result, conclusions drawn from these subgroup analyses should be interpreted with caution. Future research with larger, more comprehensive datasets is essential to validate and strengthen these findings. Third, the ICIs analyzed in this meta-analysis comprised different agents, including pembrolizumab, durvalumab, cadonilimab, and atezolizumab, which could account for observed differences in outcomes. Future research should aim to conduct targeted analyses focusing on the safety and efficacy of individual ICIs in CC once sufficient studies and high-quality evidence become available. Fourth, the absence of essential biomarker data, such as HPV status, MSI testing, and detailed TMB information, represents a significant limitation for conducting robust subgroup analyses. This lack of biomarker insights significantly limits the ability to identify molecular predictors of response to ICIs. Fifth, TSA indicated that additional RCTs with larger sample sizes are needed to corroborate our findings regarding all-cause AEs of any grade.

## 5 Conclusion

Our study revealed that adding ICIs to standard CT or CRT produced notable clinical benefit in patients with advanced or recurrent CC, reflected by significant improvements in PFS, OS, and ORR. Testing PD-L1 status could serve as a useful predictive biomarker to identify those most likely to obtain greater benefit from ICI-containing combination regimens. Notably, the addition of ICIs did not increase the risk of all-cause AEs of any grade. However, an increased incidence of grade 3–5 AEs was observed, underscoring the need for careful patient selection and vigilant toxicity management.

## Data Availability

The original contributions presented in the study are included in the article/**Supplementary Material**. Further inquiries can be directed to the corresponding author.
