# Supplementary material for: Efficacy and safety of adding immune checkpoint inhibitors to standard chemotherapy or chemoradiotherapy for advanced or recurrent cervical cancer: a meta-analysis
Source: Front Immunol. 2026 Mar 5;17:1780791. doi: 10.3389/fimmu.2026.1780791 (PMC12999943; doi:10.3389/fimmu.2026.1780791)
Supplement: Supplementary file 1 [file DataSheet1.docx]

**PubMed 1316**

#1 (immune checkpoint inhibitors) OR (immune checkpoint inhibitor) OR (immune checkpoint blockade) OR (immune checkpoint therapy) OR (PD-1) OR (programmed death 1) OR (PD-L1) OR (programmed death-ligand 1) OR (CTLA-4) OR (cytotoxic T-lymphocyte-associated protein 4) OR (pembrolizumab) OR (nivolumab) OR (cemiplimab) OR (dostarlimab) OR (atezolizumab) OR (durvalumab) OR (avelumab) OR (ipilimumab) OR (tremelimumab) OR (cadonilimab) OR (AK104) OR (toripalimab) OR (camrelizumab) OR (tislelizumab) OR (sintilimab)

#2 (uterine cervical neoplasms) OR (cervical cancer) OR (cervical carcinoma) OR (cervical neoplasm*) OR (cancer of the cervix) OR (cervix cancer) OR (uterine cervical neoplasm*)

#3 (randomized controlled trial) OR (randomized) OR (randomised) OR (randomly) OR (placebo) OR (clinical trial)

#4 #1 AND #2 AND #3

**Web of Science 524**

#1 TS=((immune checkpoint inhibitors) OR (immune checkpoint inhibitor) OR (immune checkpoint blockade) OR (immune checkpoint therapy) OR (PD-1) OR (programmed death 1) OR (PD-L1) OR (programmed death-ligand 1) OR (CTLA-4) OR (cytotoxic T-lymphocyte-associated protein 4) OR (pembrolizumab) OR (nivolumab) OR (cemiplimab) OR (dostarlimab) OR (atezolizumab) OR (durvalumab) OR (avelumab) OR (ipilimumab) OR (tremelimumab) OR (cadonilimab) OR (AK104) OR (toripalimab) OR (camrelizumab) OR (tislelizumab) OR (sintilimab))

#2 TS=((uterine cervical neoplasms) OR (cervical cancer) OR (cervical carcinoma) OR (cervical neoplasm*) OR (cancer of the cervix) OR (cervix cancer) OR (uterine cervical neoplasm*))

#3 TS=((randomized controlled trial) OR (randomized) OR (randomised) OR (randomly) OR (placebo) OR (clinical trial))

#4 #1 AND #2 AND #3

**Embase 1336**

#1 'immune checkpoint inhibitors'/exp OR 'immune checkpoint inhibitor'/exp OR 'immune checkpoint blockade'/exp OR 'immune checkpoint therapy'/exp OR 'pd 1'/exp OR 'programmed death 1'/exp OR 'pd l1' OR 'programmed death-ligand 1'/exp OR 'ctla 4'/exp OR 'cytotoxic t-lymphocyte-associated protein 4' OR 'pembrolizumab'/exp OR 'nivolumab'/exp OR 'cemiplimab'/exp OR 'dostarlimab'/exp OR 'atezolizumab'/exp OR 'durvalumab'/exp OR 'avelumab'/exp OR 'ipilimumab'/exp OR 'tremelimumab'/exp OR 'cadonilimab'/exp OR 'ak104'/exp OR 'toripalimab'/exp OR 'camrelizumab'/exp OR 'tislelizumab'/exp OR 'sintilimab'/exp

#2 'uterine cervical neoplasms'/exp OR 'cervical cancer'/exp OR 'cervical carcinoma'/exp OR 'cervical neoplasm*' OR 'cancer of the cervix'/exp OR 'cervix cancer'/exp OR 'uterine cervical neoplasm*'

#3 'randomized controlled trial'/exp OR randomized OR randomised OR randomly OR 'placebo'/exp OR 'clinical trial'/exp

#4 #1 AND #2 AND #3

**The Cochrane Library 337**

#1 All Text=((immune checkpoint inhibitors) OR (immune checkpoint inhibitor) OR (immune checkpoint blockade) OR (immune checkpoint therapy) OR (PD-1) OR (programmed death 1) OR (PD-L1) OR (programmed death-ligand 1) OR (CTLA-4) OR (cytotoxic T-lymphocyte-associated protein 4) OR (pembrolizumab) OR (nivolumab) OR (cemiplimab) OR (dostarlimab) OR (atezolizumab) OR (durvalumab) OR (avelumab) OR (ipilimumab) OR (tremelimumab) OR (cadonilimab) OR (AK104) OR (toripalimab) OR (camrelizumab) OR (tislelizumab) OR (sintilimab))

#2 All Text=((uterine cervical neoplasms) OR (cervical cancer) OR (cervical carcinoma) OR (cervical neoplasm*) OR (cancer of the cervix) OR (cervix cancer) OR (uterine cervical neoplasm*))

#3 All Text=((randomized controlled trial) OR (randomized) OR (randomised) OR (randomly) OR (placebo) OR (clinical trial))

#4 #1 AND #2 AND #3
