# Supplementary material for: Efficacy and safety of adding immune checkpoint inhibitors to standard chemotherapy or chemoradiotherapy for advanced or recurrent cervical cancer: a meta-analysis
Source: Front Immunol. 2026 Mar 5;17:1780791. doi: 10.3389/fimmu.2026.1780791 (PMC12999943; doi:10.3389/fimmu.2026.1780791)

**FIGURE S1** Subgroup analysis of progression-free survival based on therapeutic targets. (A) Programmed cell death protein 1 (PD-1); (B) Programmed death-ligand 1 (PD-L1); (C) PD-1 and cytotoxic T-lymphocyte-associated protein 4 (CTLA-4).


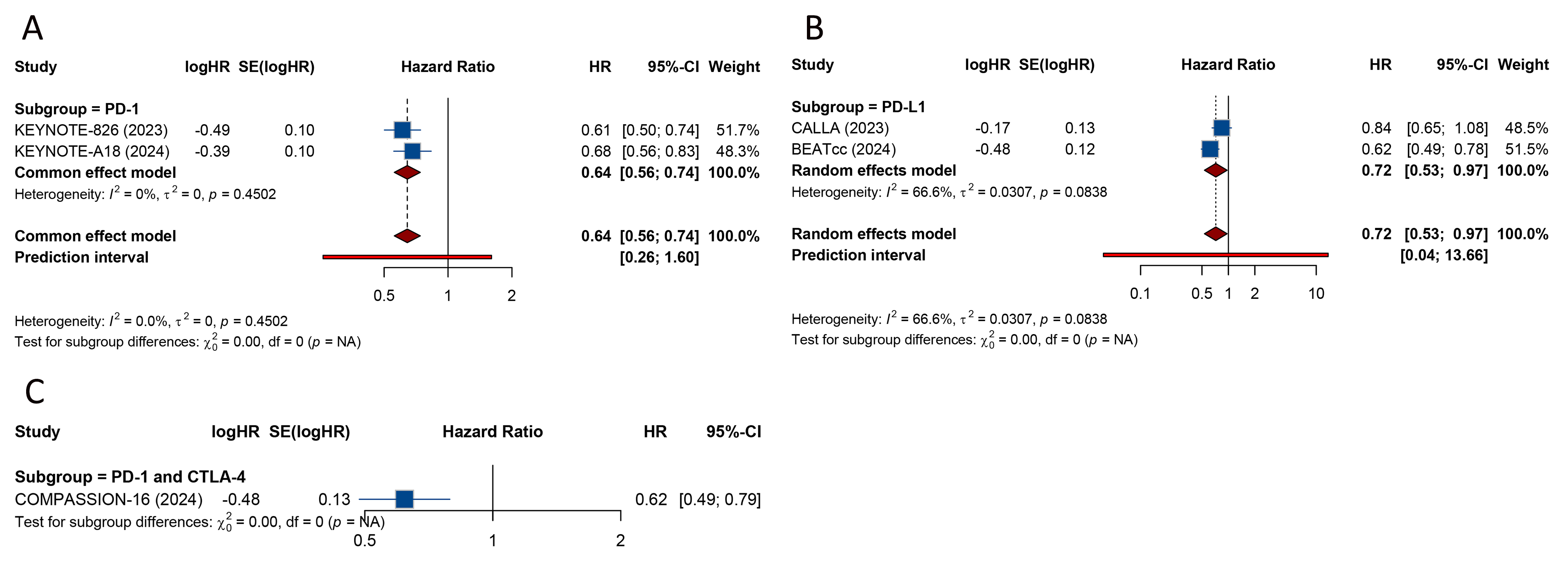


**FIGURE S2** Subgroup analysis of progression-free survival according to the age of patients. (A) < 65 years; (B) ≥ 65 years.


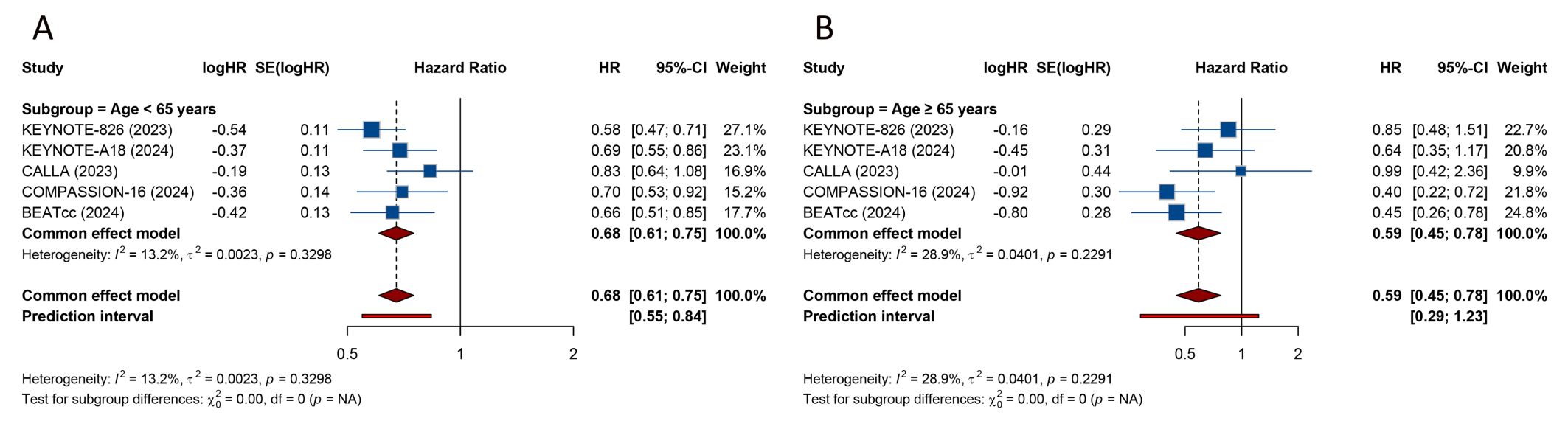


**FIGURE S3** Subgroup analysis of progression-free survival according to patient race. (A) White; (B) Asian.


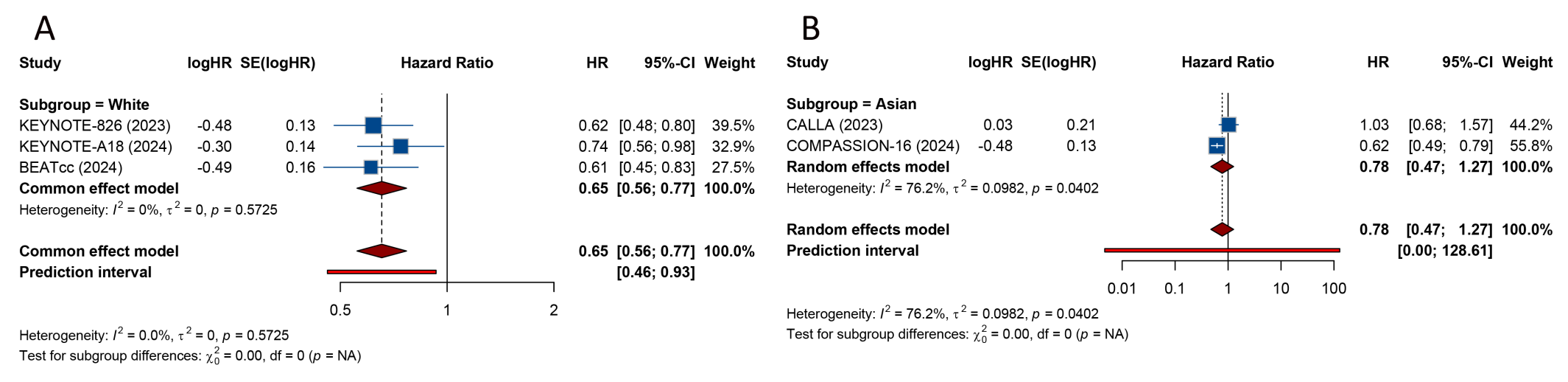


**FIGURE S4** Subgroup analysis of progression-free survival according to disease status. (A) Metastatic; (B) Non-metastatic.


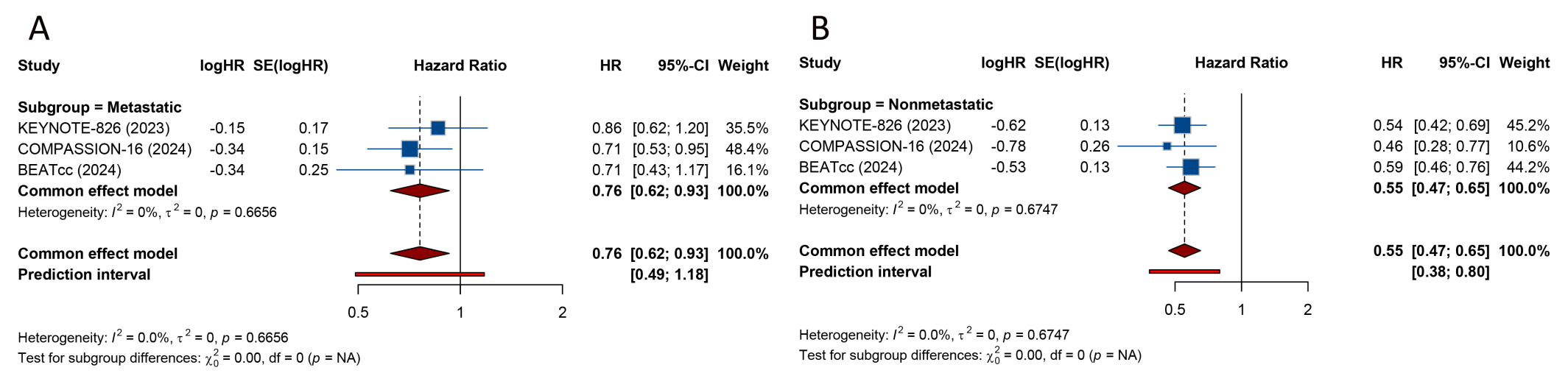


**FIGURE S5** Subgroup analysis of progression-free survival according to Eastern Cooperative Oncology Group (ECOG) performance status (PS). (A) ECOG PS of 0; (B) ECOG PS of 1.


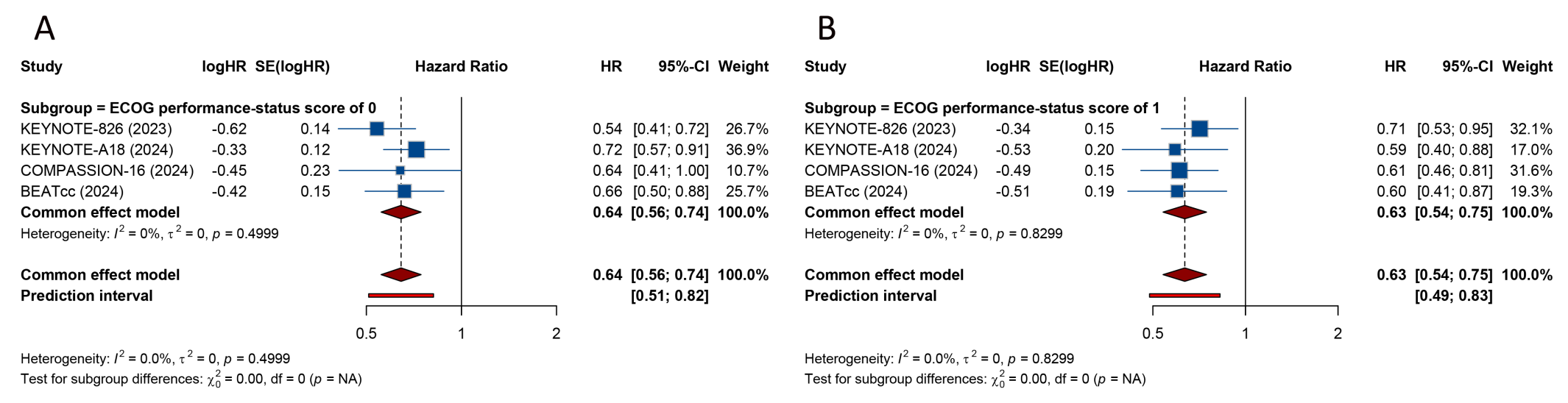


**FIGURE S6** Subgroup analysis of progression-free survival according to PD-L1 combined positive score. (A) <1; (B) 1 to <10; (C) ≥10.


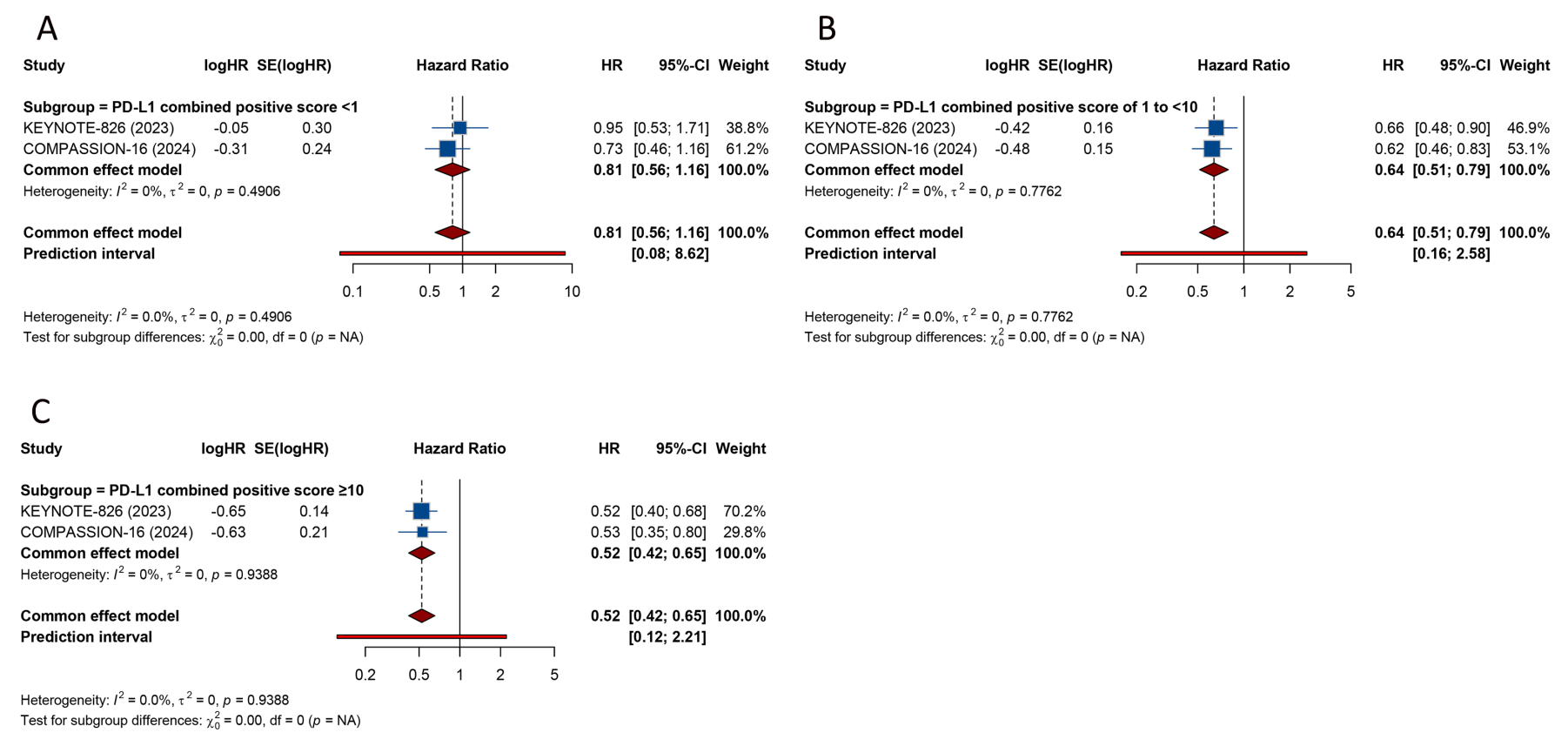


**FIGURE S7** Subgroup analysis of progression-free survival according to concomitant bevacizumab. (A) Yes; (B) No.


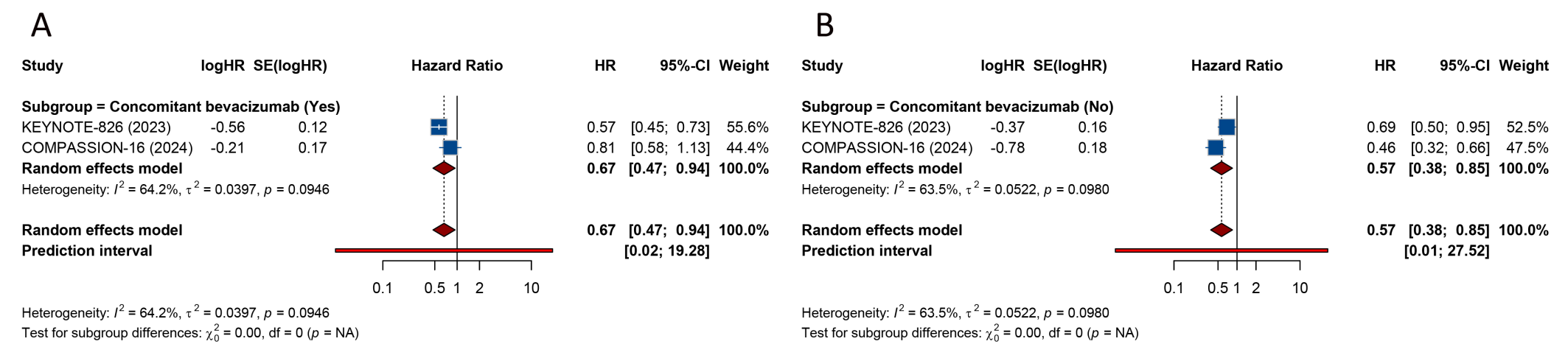


**FIGURE S8** Subgroup analysis of progression-free survival according to chemotherapy backbone. (A) Cisplatin; (B) Carboplatin.


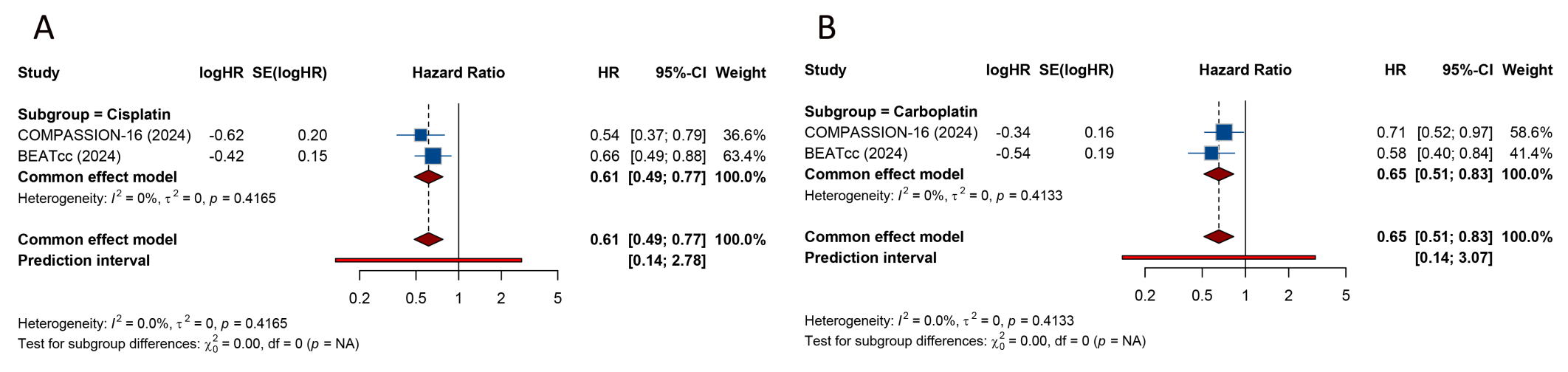


**FIGURE S9** Subgroup analysis of progression-free survival according to previous chemoradiotherapy. (A) Yes; (B) No.


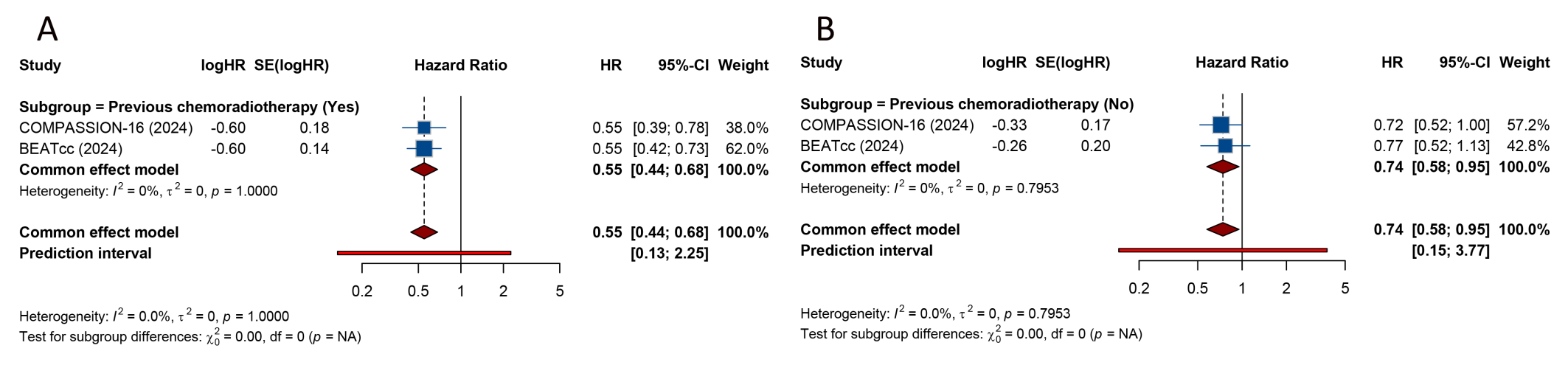


**FIGURE S10** Subgroup analysis of overall survival based on therapeutic targets. (A) Programmed cell death protein 1 (PD-1); (B) Programmed death-ligand 1 (PD-L1); (C) PD-1 and cytotoxic T-lymphocyte-associated protein 4 (CTLA-4).


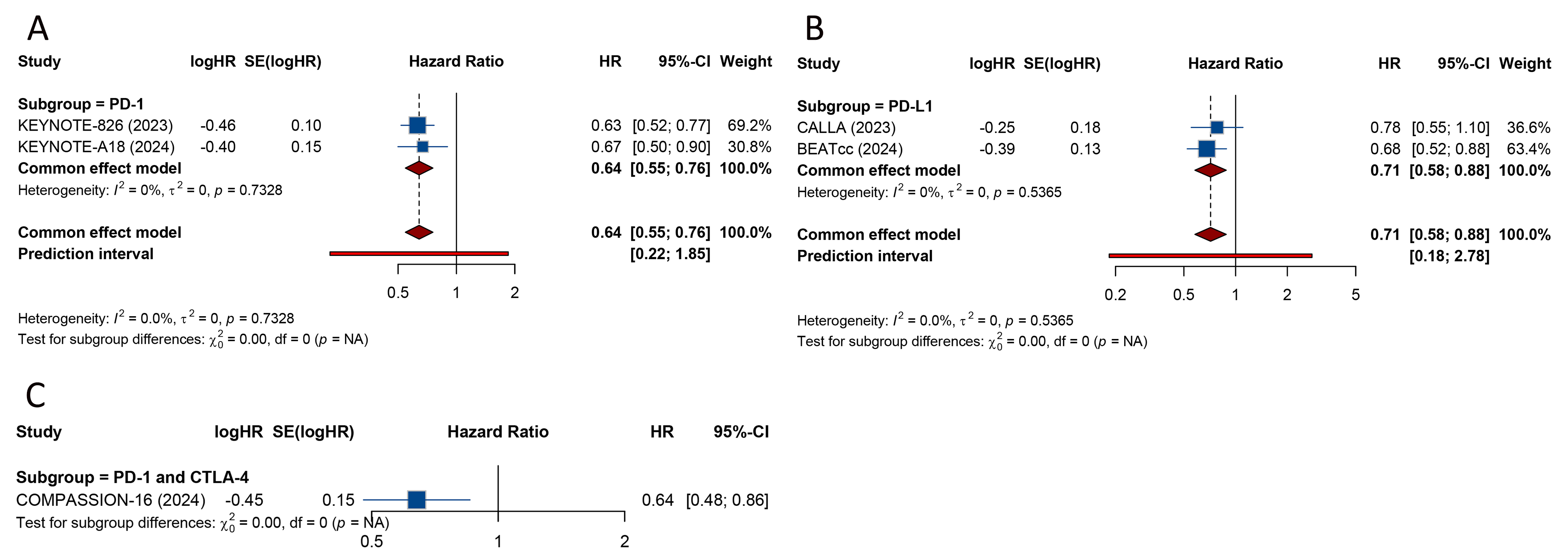


**FIGURE S11** Subgroup analysis of overall survival according to the age of patients. (A) < 65 years; (B) ≥ 65 years.


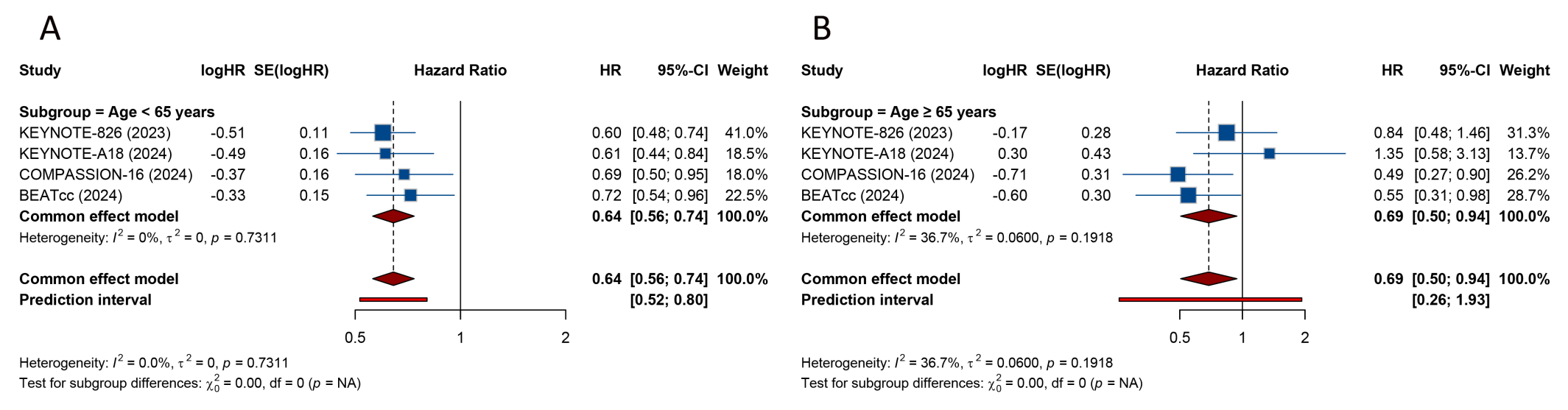


**FIGURE S12** Subgroup analysis of overall survival according to patient race. (A) White; (B) All others.


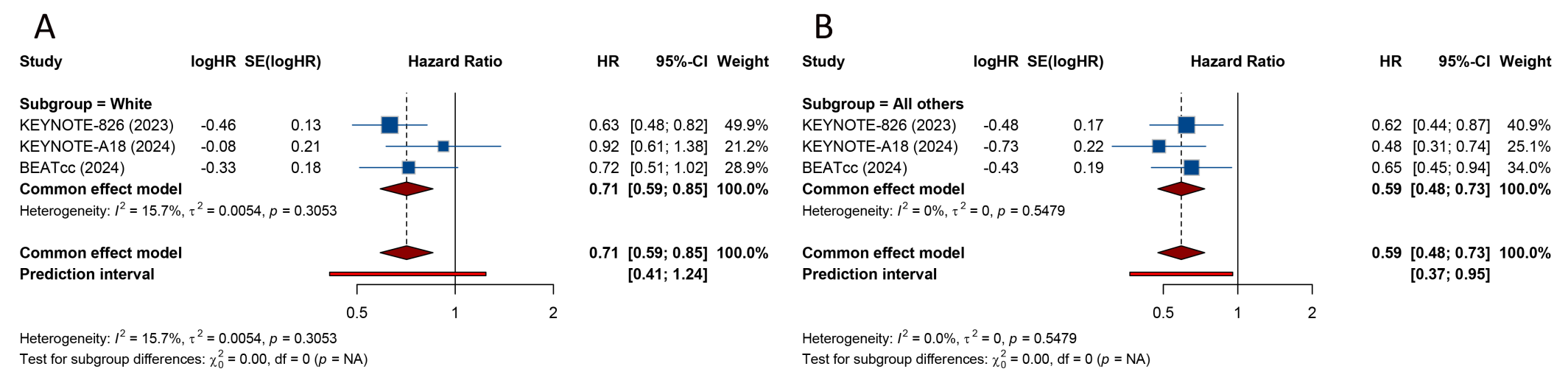


**FIGURE S13** Subgroup analysis of overall survival according to disease status. (A) Metastatic; (B) Non-metastatic.


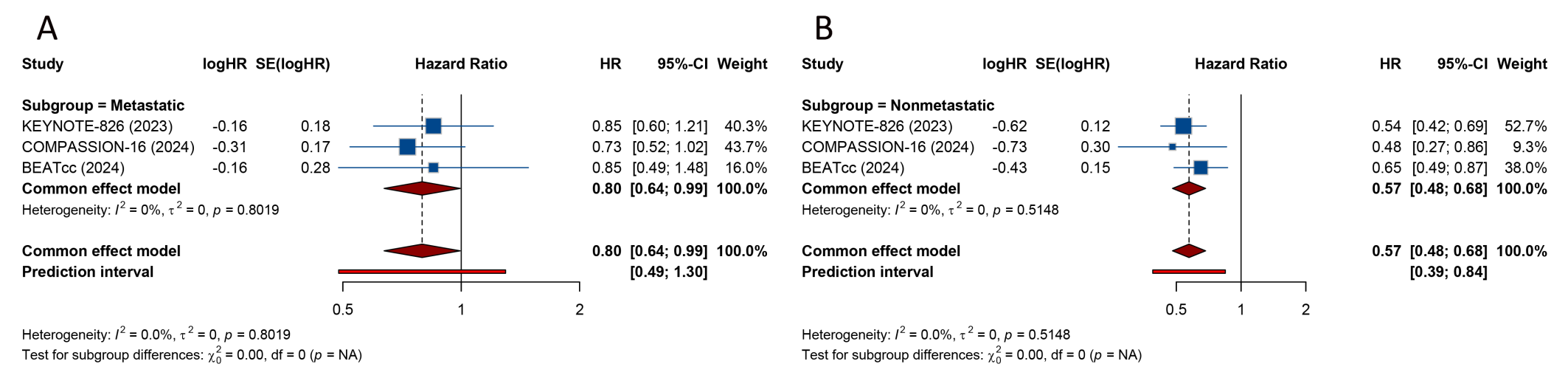


**FIGURE S14** Subgroup analysis of overall survival according to Eastern Cooperative Oncology Group (ECOG) performance status (PS). (A) ECOG PS of 0; (B) ECOG PS of 1.


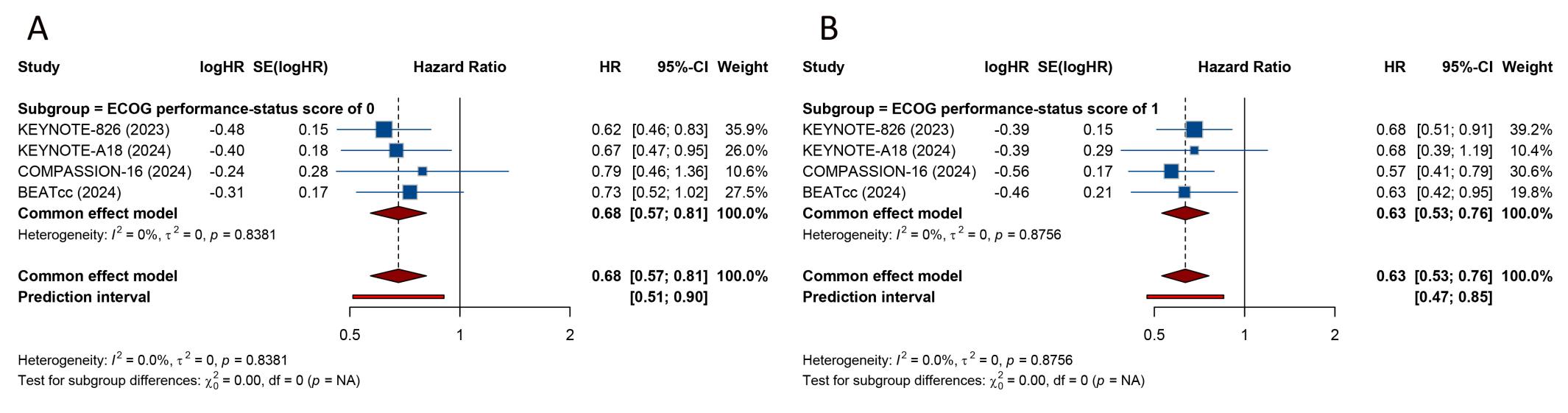


**FIGURE S15** Subgroup analysis of overall survival according to PD-L1 combined positive score. (A) <1; (B) 1 to <10; (C) ≥10.


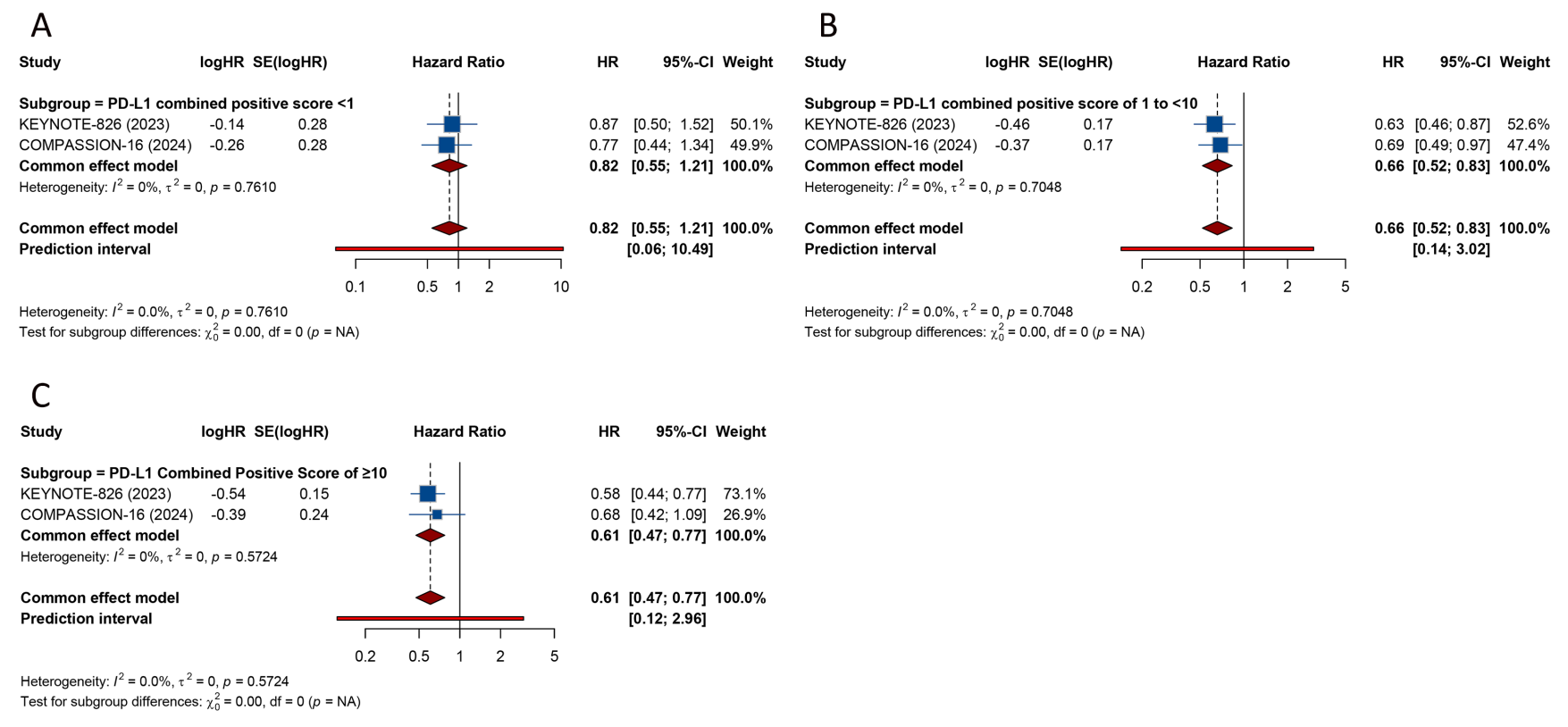


**FIGURE S16** Subgroup analysis of overall survival according to concomitant bevacizumab. (A) Yes; (B) No.


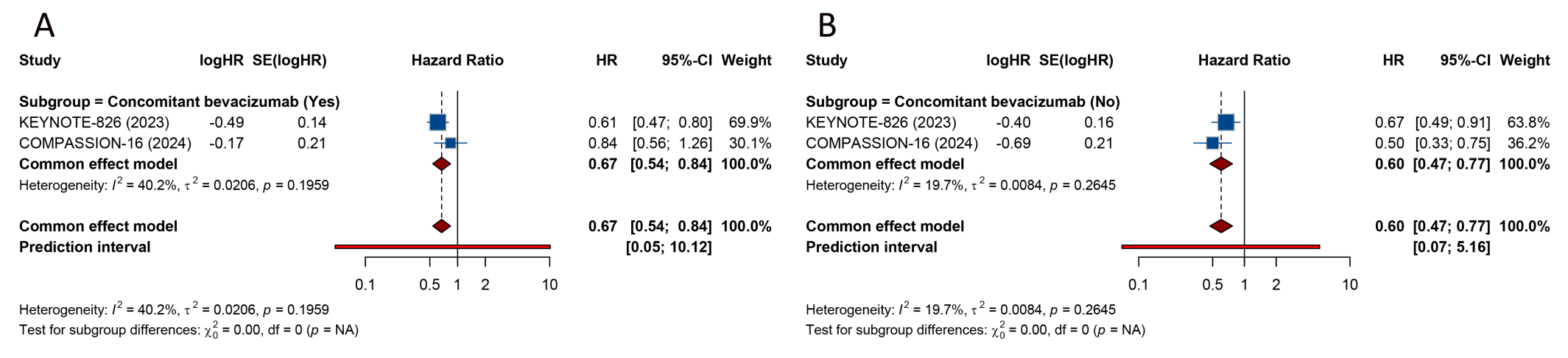


**FIGURE S17** Subgroup analysis of overall survival according to chemotherapy backbone. (A) Cisplatin; (B) Carboplatin.


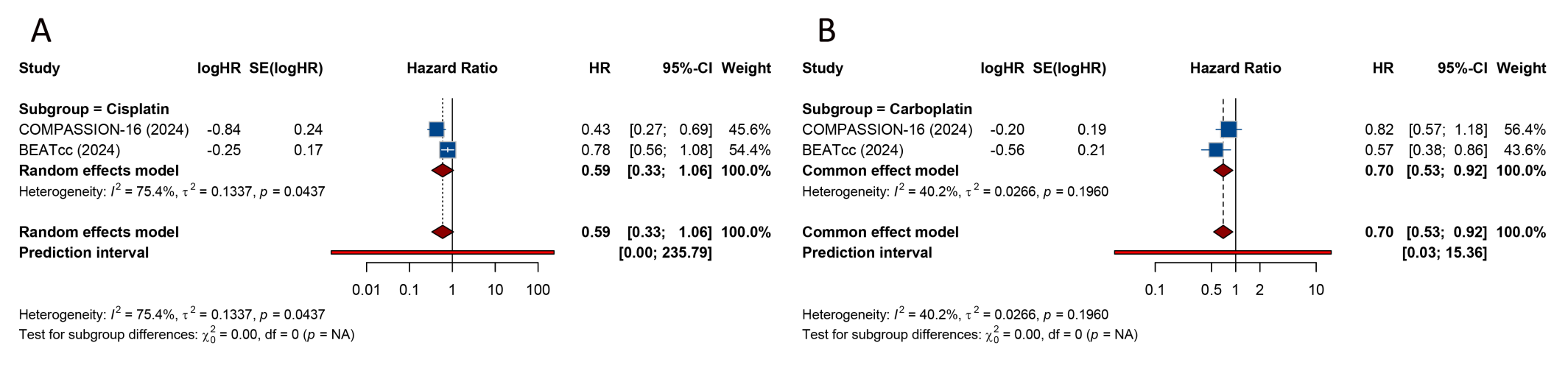


**FIGURE S18** Subgroup analysis of overall survival according to previous chemoradiotherapy. (A) Yes; (B) No.


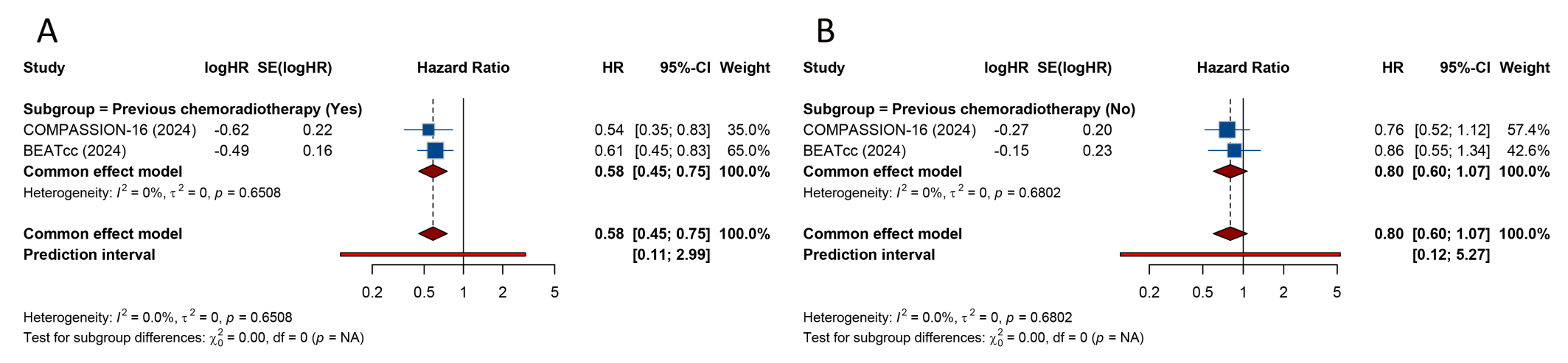


**FIGURE S19** Subgroup analysis of objective response rate based on therapeutic targets. (A) Programmed cell death protein 1 (PD-1); (B) Programmed death-ligand 1 (PD-L1); (C) PD-1 and cytotoxic T-lymphocyte-associated protein 4 (CTLA-4).


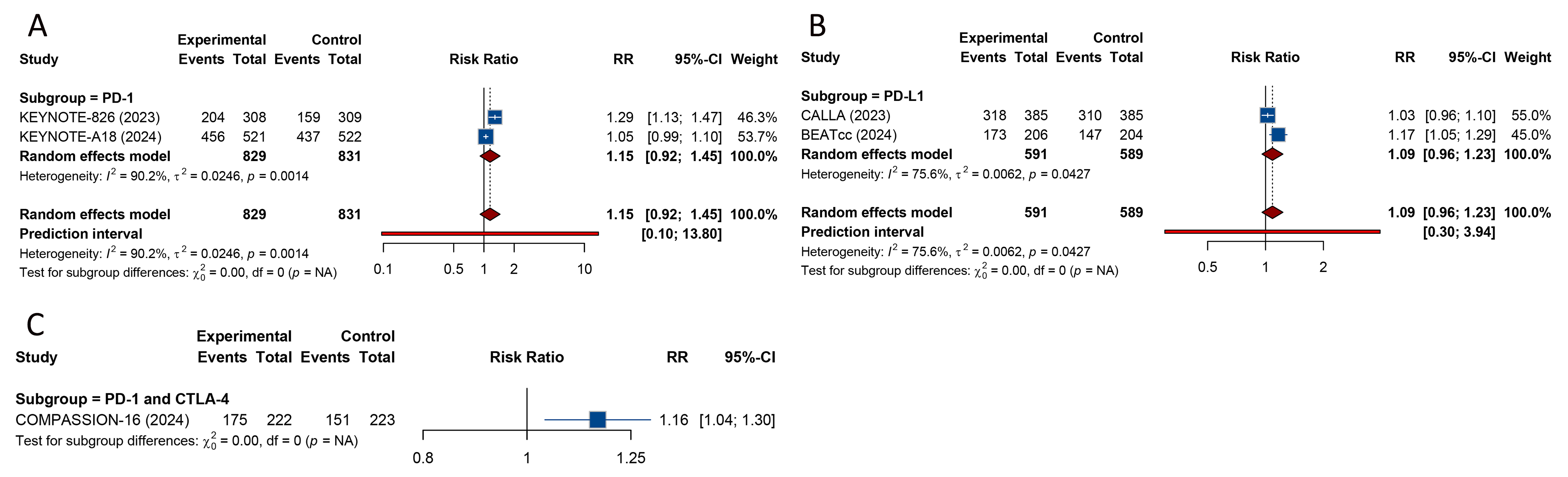


**FIGURE S20** Subgroup analysis of all-cause any grade adverse events based on therapeutic targets. (A) Programmed cell death protein 1 (PD-1); (B) Programmed death-ligand 1 (PD-L1); (C) PD-1 and cytotoxic T-lymphocyte-associated protein 4 (CTLA-4).


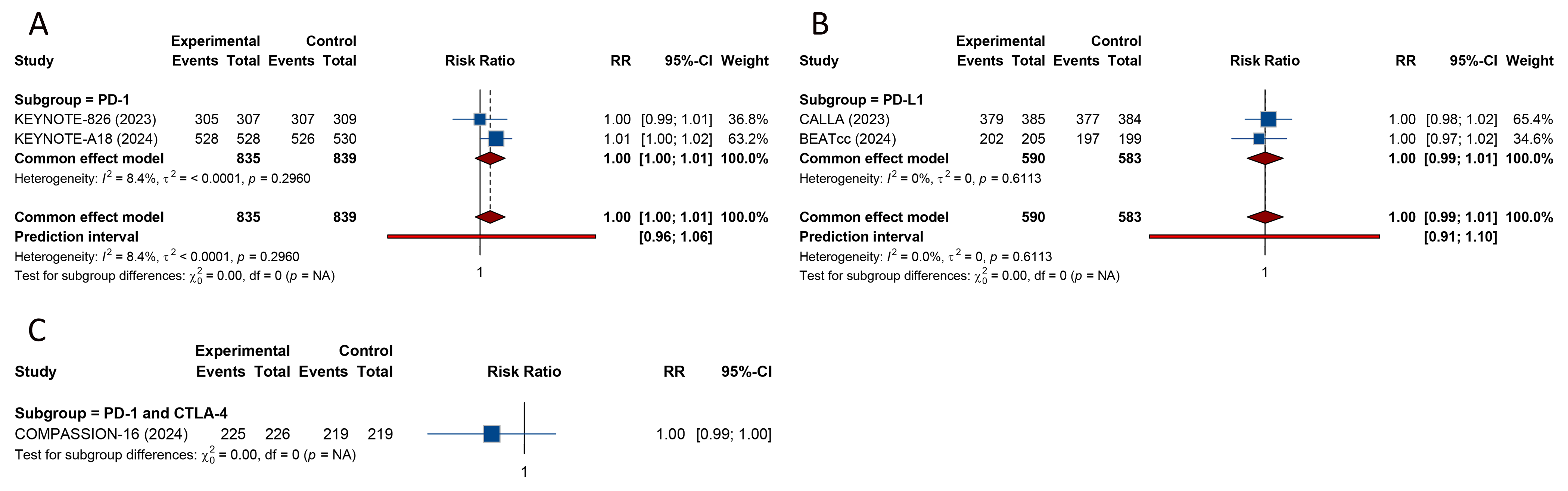


**FIGURE S21** Forest plots of specific all-cause adverse events of any grade. (A) Anemia; (B) Vomiting; (C) Diarrhea; (D) Constipation; (E) Nausea; (F) Decreased appetite; (G) Urinary tract infection.


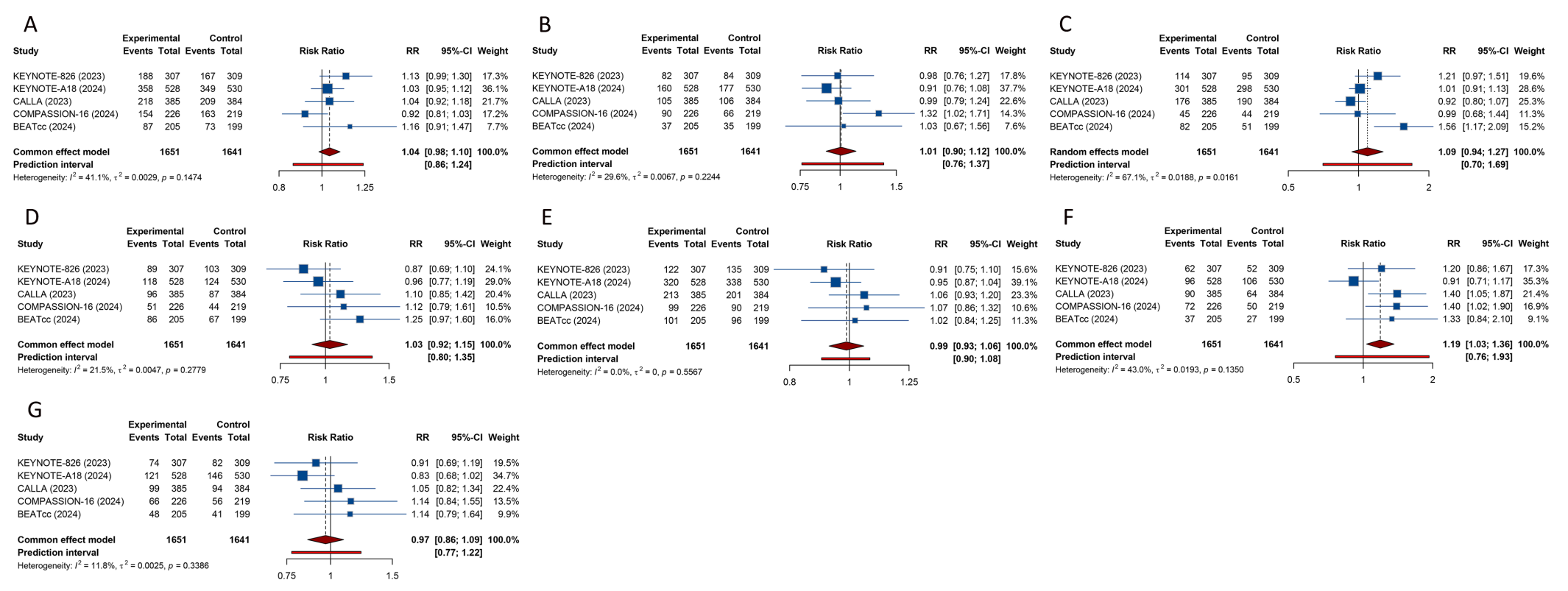


**FIGURE S22** Forest plots of specific all-cause adverse events of any grade. (A) Fatigue; (B) Hypothyroidism; (C) Neutropenia; (D) Platelet count decreased; (E) White blood cell count decreased; (F) Alanine aminotransferase increased.


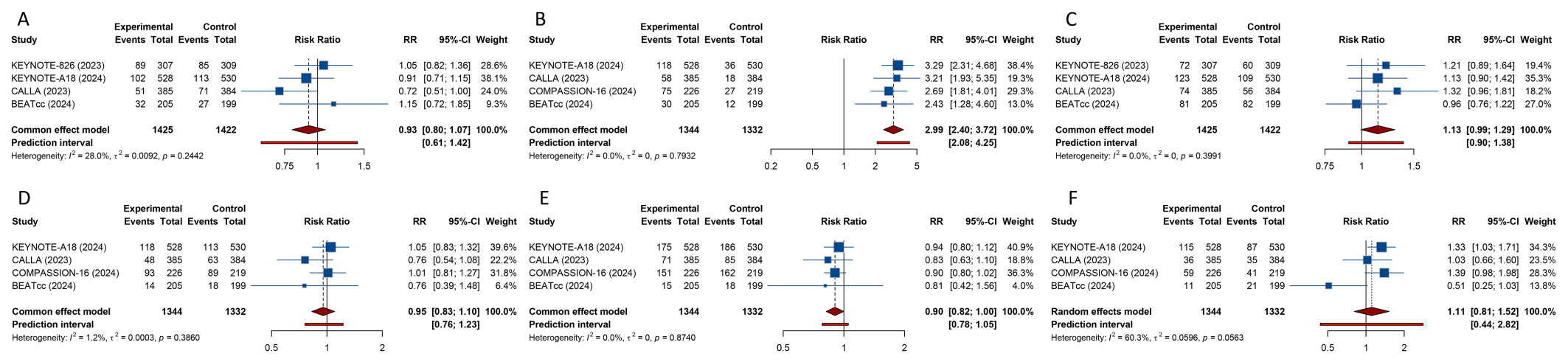


**FIGURE S23** Subgroup analysis of all-cause grade 3-5 adverse events based on therapeutic targets. (A) Programmed cell death protein 1 (PD-1); (B) Programmed death-ligand 1 (PD-L1); (C) PD-1 and cytotoxic T-lymphocyte-associated protein 4 (CTLA-4).


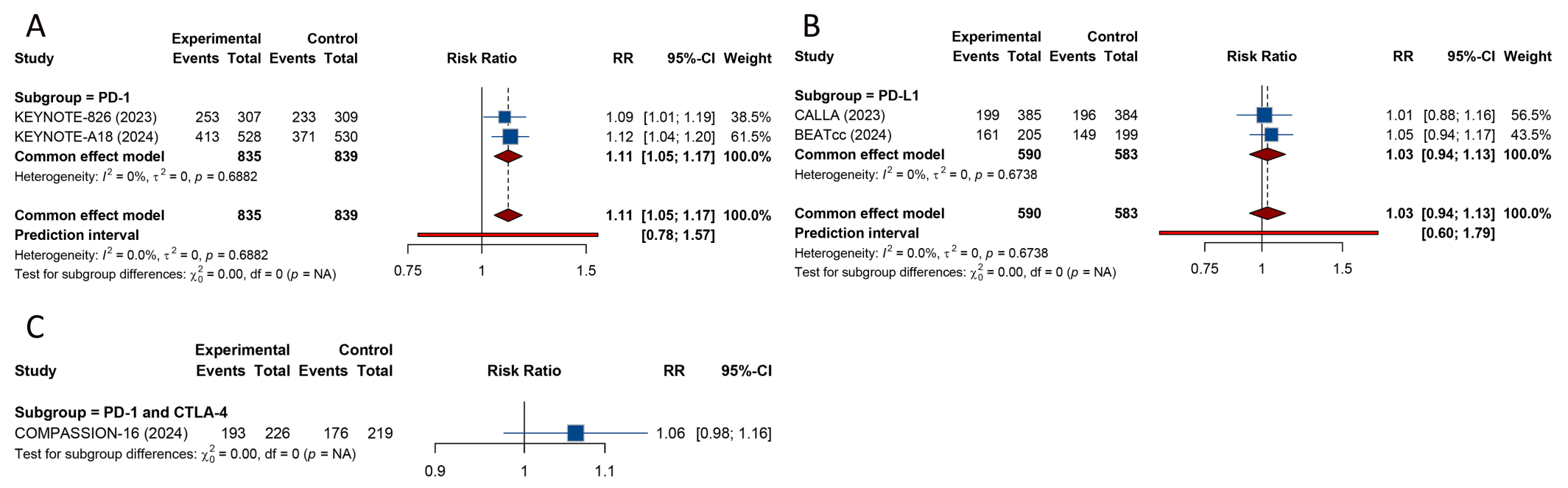


**FIGURE S24** Forest plots of specific all-cause adverse events of grade 3-5. (A) Anemia; (B) Vomiting; (C) Diarrhea; (D) Constipation; (E) Nausea; (F) Decreased appetite; (G) Urinary tract infection.


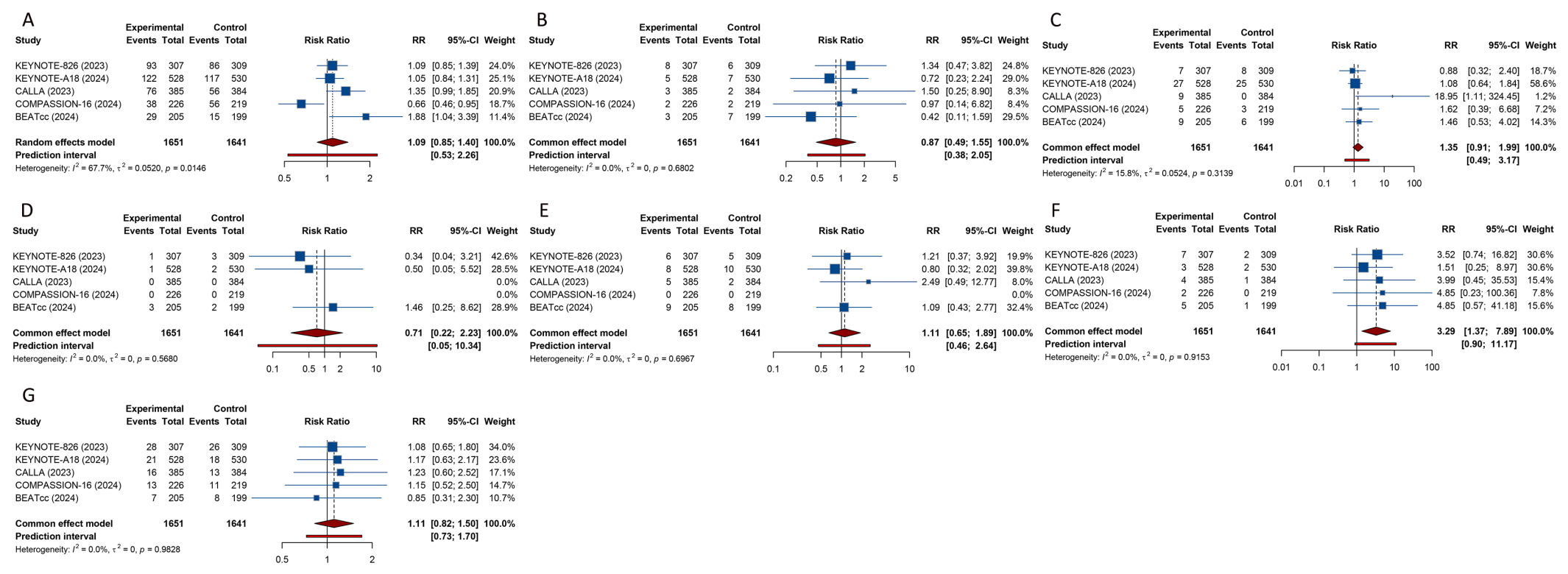


**FIGURE S25** Forest plots of specific all-cause adverse events of grade 3-5. (A) Fatigue; (B) Hypothyroidism; (C) Neutropenia; (D) Platelet count decreased; (E) White blood cell count decreased; (F) Alanine aminotransferase increased.


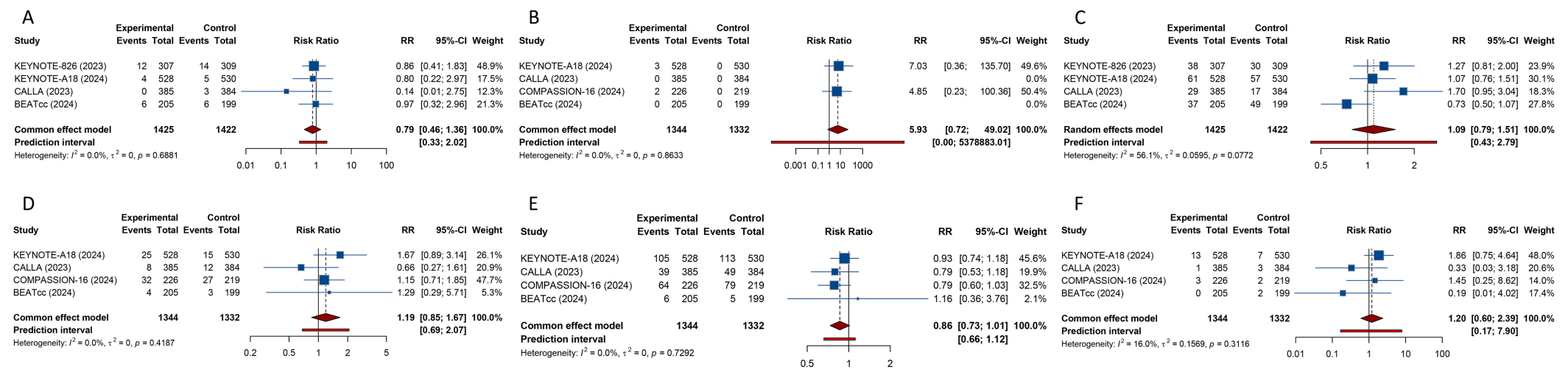


**FIGURE S26** Sensitivity analysis of adding immune checkpoint inhibitors to standard chemotherapy or chemoradiotherapy for advanced or recurrent cervical cancer. (A) Progression-free survival; (B) Overall survival; (C) Objective response rate; (D) All-cause adverse events (AEs) of any grade; (E) All-cause AEs of grade 3-5.


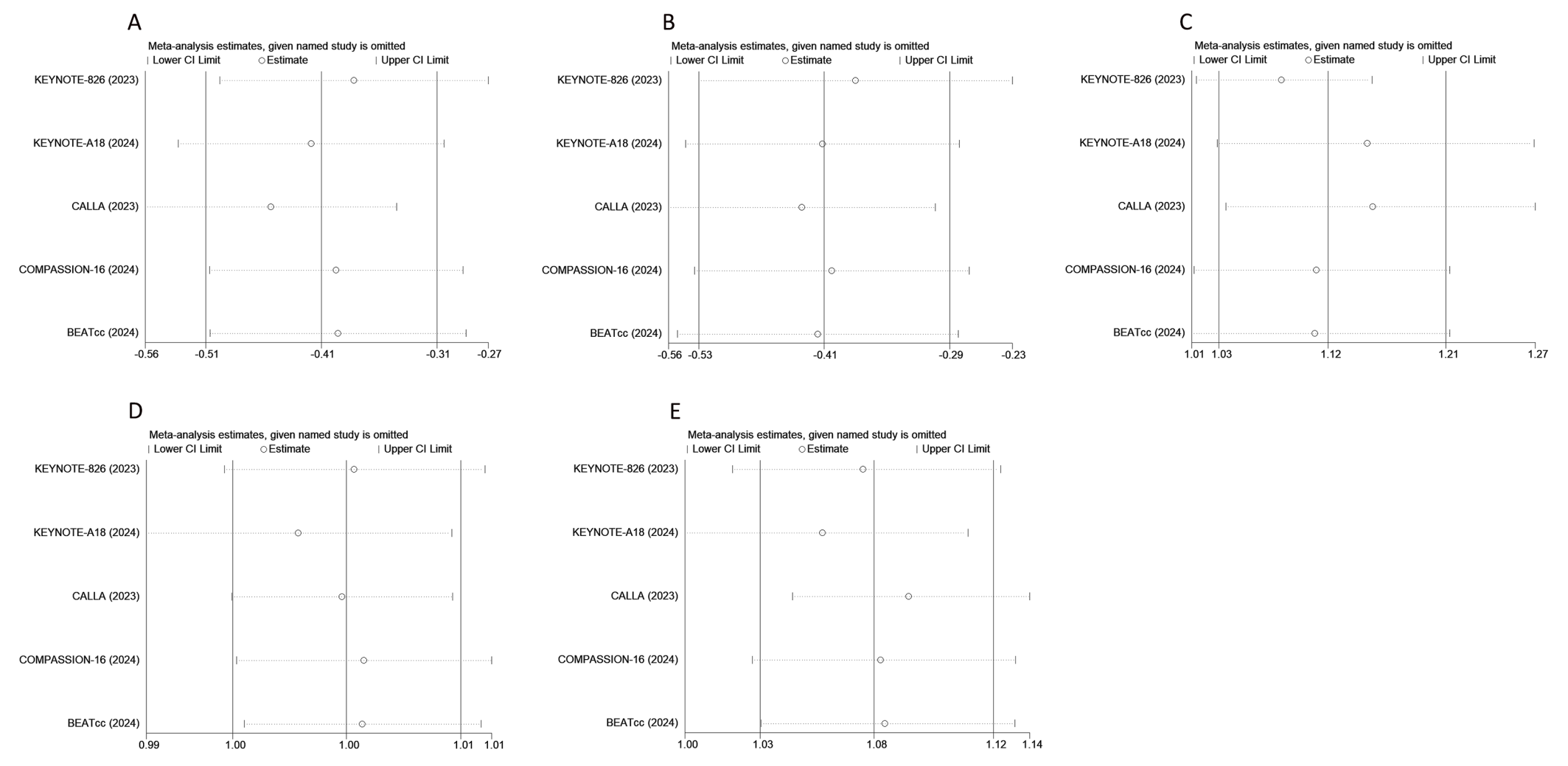


**FIGURE S27** Funnel plots of adding immune checkpoint inhibitors to standard chemotherapy or chemoradiotherapy for advanced or recurrent cervical cancer. (A) Progression-free survival; (B) Overall survival; (C) Objective response rate; (D) All-cause adverse events (AEs) of any grade; (E) All-cause AEs of grade 3-5.


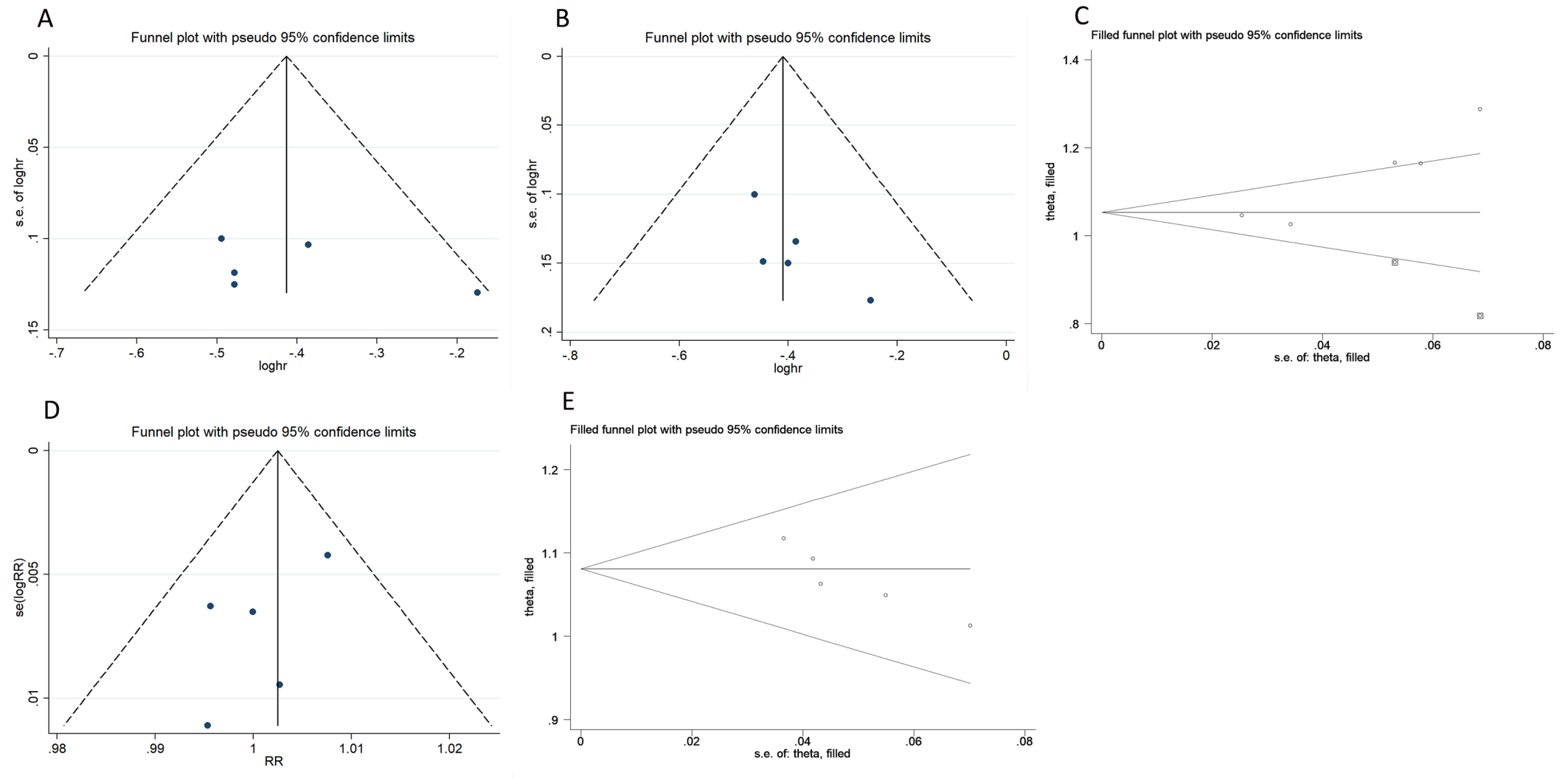

Supplement: Supplementary file 2 [file DataSheet2.docx]
