# Supplementary material for: Efficacy and safety of adding immune checkpoint inhibitors to standard chemotherapy or chemoradiotherapy for advanced or recurrent cervical cancer: a meta-analysis
Source: Front Immunol. 2026 Mar 5;17:1780791. doi: 10.3389/fimmu.2026.1780791 (PMC12999943; doi:10.3389/fimmu.2026.1780791)
Supplement: Supplementary file 3 [file Table1.docx]

| TABLE S1 Quality assessment of the included RCTs by modified Jadad scale. | | | | | | |
| --- | --- | --- | --- | --- | --- | --- |
| Trial | Randomization | Randomization concealment | Double blind | Withdrawals and dropouts | Score | Study quality |
| KEYNOTE-A18 | 2 | 2 | 2 | 1 | 7 | High |
| CALLA | 2 | 2 | 2 | 1 | 7 | High |
| COMPASSION-16 | 2 | 2 | 2 | 1 | 7 | High |
| KEYNOTE-826 | 2 | 2 | 2 | 1 | 7 | High |
| BEATcc | 2 | 2 | 0 | 1 | 5 | High |
